# Safety and Proof-of-Concept Study of Oral QLT091001 in Retinitis Pigmentosa Due to Inherited Deficiencies of Retinal Pigment Epithelial 65 Protein (RPE65) or Lecithin:Retinol Acyltransferase (LRAT)

**DOI:** 10.1371/journal.pone.0143846

**Published:** 2015-12-10

**Authors:** Hendrik P. N. Scholl, Anthony T. Moore, Robert K. Koenekoop, Yuquan Wen, Gerald A. Fishman, L. Ingeborgh van den Born, Ava Bittner, Kristen Bowles, Emily C. Fletcher, Frederick T. Collison, Gislin Dagnelie, Simona Degli Eposti, Michel Michaelides, David A. Saperstein, Ronald A. Schuchard, Claire Barnes, Wadih Zein, Ditta Zobor, David G. Birch, Janine D. Mendola, Eberhart Zrenner, RET IRD 01 Study Group

**Affiliations:** 1 Wilmer Eye Institute, Johns Hopkins University, Baltimore, MD, United States of America; 2 Moorfields Eye Hospital and Institute of Ophthalmology, University College London, London, United Kingdom; 3 Dept. of Ophthalmology, University of California San Francisco, San Francisco, CA, United States of America; 4 McGill University Health Centre, Montreal, Quebec, Canada; 5 Retina Foundation of the Southwest, Dallas, TX, United States of America; 6 Baylor Visual Function Center, Baylor University Medical Center, Dallas, TX, United States of America; 7 Chicago Lighthouse, Pangere Center for Inherited Retinal Diseases, Chicago, IL, United States of America; 8 Rotterdam Eye Hospital and Ophthalmic Institute, Rotterdam, The Netherlands; 9 Nova Southeastern University, College of Optometry, Fort Lauderdale, FL, United States of America; 10 College of Optometry, University of Houston, Houston, TX, United States of America; 11 Gloucester Royal NHS Foundation Trust, Gloucester Royal Hospitals, Gloucester, United Kingdom; 12 Vitreoretinal Associates of Washington, Seattle, WA, United States of America; 13 QLT Inc., Vancouver, Canada; 14 Stanford Univ School of Medicine, Palo Alto, CA, United States of America; 15 VA Palo Alto Health Care System, Palo Alto, CA, United States of America; 16 Division of Epidemiology and Clinical Research, National Eye Institute, National Institutes of Health, Bethesda, MD, United States of America; 17 Institute for Ophthalmic Research, Center for Ophthalmology, University of Tübingen, Tübingen, Germany; 18 Ophthalmology, UT Southwestern, Dallas, TX, United States of America; International University of Health and Welfare, JAPAN

## Abstract

Restoring vision in inherited retinal degenerations remains an unmet medical need. In mice exhibiting a genetically engineered block of the visual cycle, vision was recently successfully restored by oral administration of 9-*cis*-retinyl acetate (QLT091001). Safety and visual outcomes of a once-daily oral dose of 40 mg/m^2^/day QLT091001 for 7 consecutive days was investigated in an international, multi-center, open-label, proof-of-concept study in 18 patients with *RPE65-* or *LRAT*-related retinitis pigmentosa. Eight of 18 patients (44%) showed a ≥20% increase and 4 of 18 (22%) showed a ≥40% increase in functional retinal area determined from Goldmann visual fields; 12 (67%) and 5 (28%) of 18 patients showed a ≥5 and ≥10 ETDRS letter score increase of visual acuity, respectively, in one or both eyes at two or more visits within 2 months of treatment. In two patients who underwent fMRI, a significant positive response was measured to stimuli of medium contrast, moving, pattern targets in both left and right hemispheres of the occipital cortex. There were no serious adverse events. Treatment-related adverse events were transient and the most common included headache, photophobia, nausea, vomiting, and minor biochemical abnormalities. Measuring the outer segment length of the photoreceptor layer with high-definition optical coherence tomography was highly predictive of treatment responses with responders having a significantly larger baseline outer segment thickness (11.7 ± 4.8 μm, mean ± 95% CI) than non-responders (3.5 ± 1.2 μm). This structure-function relationship suggests that treatment with QLT091001 is more likely to be efficacious if there is sufficient photoreceptor integrity.

**Trial Registration:**

ClinicalTrials.gov NCT01014052

## Introduction

Inherited retinal degenerations affect as many as 2 million, or 1 in 2000 people worldwide [1,2]. They are caused by genetic defects that result in the dysfunction, degeneration and/or maldevelopment of photoreceptors and/or the retinal pigment epithelium (RPE) [3]. Inherited retinal degenerations remain untreatable to date (with the exception of some rare retinal degenerations secondary to abnormalities in systemic metabolism such as Refsum disease, abetalipoproteinemia and gyrate atrophy).

Retinitis pigmentosa (RP) is the most common inherited retinal degeneration, with a prevalence of approximately 1 in 3500 [4,5]. The term RP describes a group of rod-cone dystrophies that are genetically heterogeneous. Affected patients frequently experience night blindness in the early phase of the disease, followed by loss of mid-peripheral field of vision with progression towards the center of fixation [6]. At the cellular level, mutations causing RP initially affect primarily the rod photoreceptors. In later stages, the disease inexorably affects the cone photoreceptors, which may eventually cause complete blindness. Genes mutated in RP encode proteins that are involved in multiple mechanisms and pathways, including the phototransduction cascade, maintenance of photoreceptor structure, gene transcription and ciliary function. In a small group of patients (5%), RP is caused by a defect in the retinol metabolism caused by genetic defects in RPE cells, where normally a continuous supply of the 11-*cis*-retinal chromophore is produced by the visual cycle to allow for photon-capture and the initiation of vision [7]. Two key enzymes of the visual cycle are the retinoid isomerase encoded by the RPE-specific protein 65 kDa (*RPE65*) gene and the lecithin:retinol acyltransferase (LRAT) encoded by the *LRAT* gene [8]. RPE65-deficient (*rpe65*-/-) mice display a block in the visual cycle [9], which leads to an absence of 11-*cis*-retinal and rhodopsin, with severe impairment of rod photoreceptor function, and eventual retinal degeneration [9]. Similar findings were reported in *lrat*-/- mice [10]. Mutations in *RPE65* [11,12] and *LRAT* [13] cause both RP and Leber congenital amaurosis (LCA) in humans (for a review see Ref 1).

Since the missing chromophore is the cause for both the dysfunction and degeneration of photoreceptors in RPE65 and LRAT deficiency, it appeared logical to replace the missing chromophore pharmacologically. Initial experiments aimed at bypassing the biochemical defect were performed by oral delivery of 9-*cis*-retinal in *rpe65-/-* mice [14,15]. 9-*cis*-retinal, which binds to opsin to form light-sensitive iso-rhodopsin, was initially selected due to its ease of synthesis and increased stability compared to 11-*cis*-retinal. Further refinement and extensive testing identified 9-*cis*-retinyl acetate as a useful experimental compound [16]. Treatment of *rpe65*-/-mice with 9-*cis*-retinyl acetate restored light sensitivity close to levels found in wild- type mice [17]. These pre-clinical experiments paved the way for human clinical trials with the aim to explore improvements to vision. Recently, a single-center, open-label, proof-of-concept, phase 1b study using QLT091001 in 14 patients with LCA was completed; this trial indicated that QLT091001 is well tolerated and can lead to clinically meaningful improvements in vision in *RPE65-* and *LRAT*-related LCA [18]. Since RPE65 and LRAT deficiency cause both dysfunction and degeneration of photoreceptors, the potential for improvement is likely to depend on the degree of photoreceptor integrity. High-resolution spectral domain optical coherence tomography (SD-OCT) allows visualization of the various layers of the human retina *in vivo* and is particularly important for quantifying decreases in photoreceptor outer segment (OS) length in RP [19–21]. It has been shown previously that OS length is related to visual function such as visual field sensitivity in RP [22,23]. Therefore, SD-OCT was used in this study to investigate photoreceptor integrity and to quantify OS length. As functional MRI was available at one site, it was used to measure the metabolic response in the visual cortex of two patients.

The purpose of this study was to test the safety and visual outcomes of the orally administered synthetic retinoid QLT091001 (9-*cis*-retinyl acetate) in patients with *RPE65-* and *LRAT*-related RP in an international, multi-center interventional clinical trial.

## Patients and Methods

**Fig 1:**
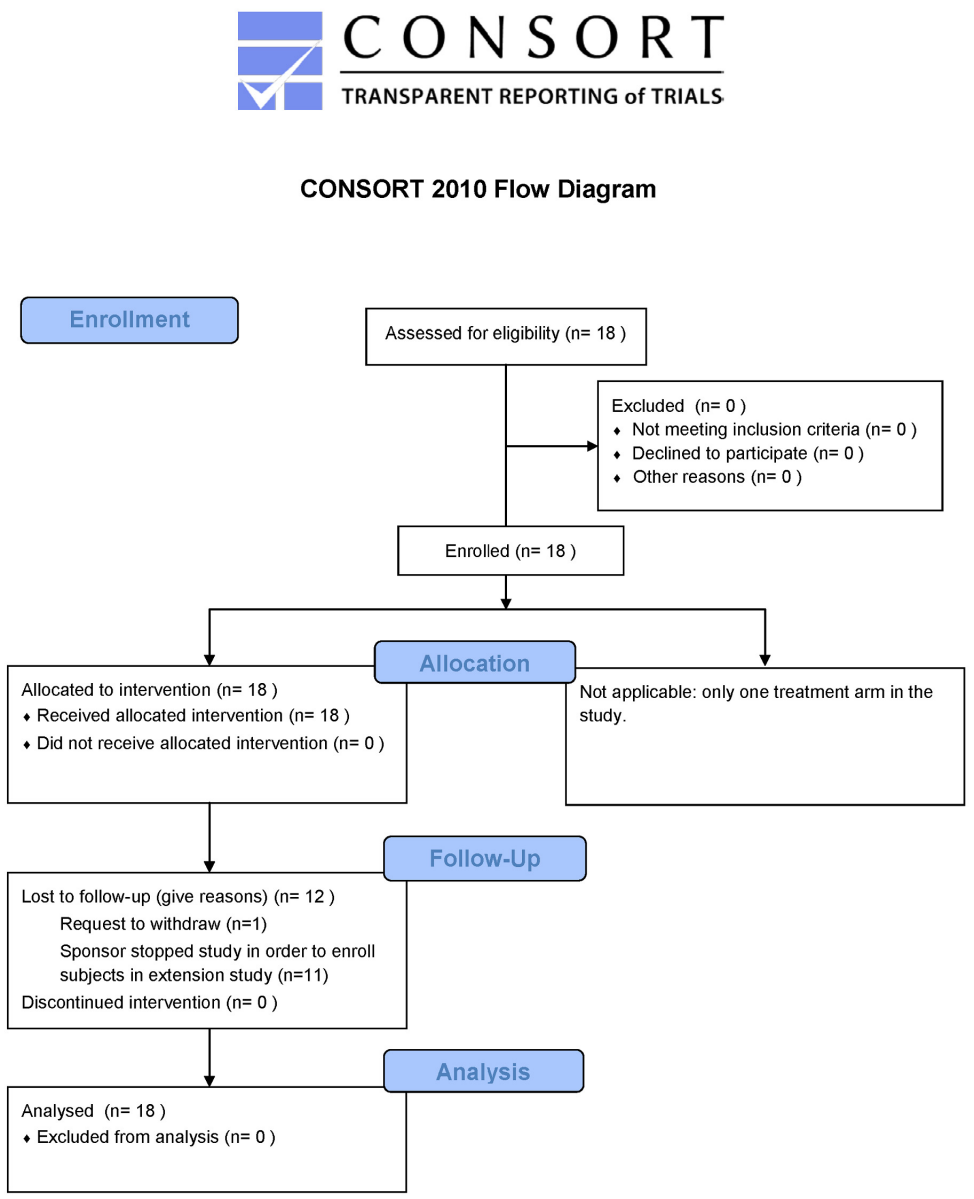
CONSORT Flow Diagram for the RET IRD 01 Study.

This was a multi-center (seven international sites, see S5 Text), open-label, proof-of-concept, phase 1b study in 18 patients with RP caused by mutation in *RPE65* or *LRAT* (registered with clinicaltrials.gov as NCT01014052). The study was approved by each center’s local Human Subjects Board Committee including the Johns Hopkins Medicine, Office of Human Subjects Research, Institutional Review Board JHM-IRB 3 (Baltimore); Montreal Children's Hospital Research Ethics Office (Montreal); Medisch Ethische Toetsings Commissie Erasmus MC (Rotterdam); North East-Sunderland Research Ethics Committee (London); Ethics Committee of the Medical Faculty of Tübingen University Hospital (Tübingen); University of Pennsylvania, Office of Regulatory Affairs (Philadelphia); Western Institutional Review Board (Chicago). Patients received a once-daily oral dose of 40 mg/m^2^/day QLT091001 for 7 consecutive days administered by a medical professional at the study center. The first patient visit occurred on 05 January 2011 and the final follow-up visit occurred on 16 August 2012. The CONSORT flow diagram is shown in Fig 1. The protocol-specified sample size of approximately 14 patients was based on the small number of patients with this condition and clinical judgment that the number was sufficient to meet the study objectives.

All patients included in the study had autosomal-recessive RP due to biallelic mutations in either the *RPE65* or *LRAT* gene confirmed in an accredited molecular genetic laboratory and were between 5 and 65 years of age (mutations in *RPE65* and *LRAT* are listed in Table 1). Patients were recruited directly by the Investigators. Patients recruited in Europe were all 18 years of age or older. Inclusion criteria included best-corrected ETDRS visual acuity of 3 letters or better (Snellen equivalent of 20/800) and the ability to undergo Goldmann visual field testing. Patients needed to be willing and able to comply with the study protocol. All participating patients were properly instructed and have indicated that they consent to participate by signing the appropriate informed consent paperwork. Written informed consent was obtained by all adult patients enrolled in the study and/or by next of kin, caretakers, or guardians on behalf of the minors/children enrolled in the study. Exclusion criteria are provided in S2 Text.

**Table 1:** Patient Characteristics, Genetic Information, Visual Field and Visual Acuity Response Profile by Patient.

Patient Number	Age (yrs)/ Gender	Gene	Zygosity (homozygous/ compound heterozygous)	cDNA	Protein	Baseline VA ETDRS Letter Score		VA Response^a^ ETDRS Letter Score		Baseline GVF Retinal area (mm^2^)		GVF Response^b^ Percent change	
						OD	OS	OD	OS	OD	OS	OD	OS
110	28/M	*LRAT*	homozygous	c.525T>A	p.Ser175Arg	70.5	59.5	8.9	10.5	42	17	68%	331%
111	41/M	*LRAT*	homozygous	c.181T>A	p.Tyr61Asp	0	1.5	No	16.0	288	288	No	No
117	6/M	*LRAT*	homozygous	c.427_428delCG	p.Arg143ValfsX3	37	39.5	14.2	11.8	263	251	52%	42%
118	11/M	*RPE65*	homozygous	c.887InsA	p.Arg296LysfsX6	40.5	19	No	No	12	4	31%	160%
201	30/M	*RPE65*	homozygous	c.1102T>C	p.Tyr368His	64	51	5.3	No	145	76	No	No
202	20/F	*LRAT*	homozygous	c.519delG	p.Ile174SerfsX12	60.5	40	No	9.5	34	25	No	No
301	37/M	*RPE65*	homozygous	c.179T>C	p.Leu60Pro	13.5	22	6.8	5.5	339	339	No	32%
302	55/F	*LRAT*	homozygous	c.40_41delGAinsTT	p.Glu14Leu	53	27.5	No	No	145	120	No	No
303	29/M	*RPE65*	homozygous	c.179T>C	p.Leu60Pro	63.5	59	No	No	204	195	No	No
304	36/F	*RPE65*	homozygous	c.179T>C	p.Leu60Pro	11.5	11.5	No	No	30	39	No	No
401	28/F	*RPE65*	homozygous	c.304G>T	p.Glu102X	0	0	1.0^c^	2.5 c	3	3	No	No
402	30/F	*RPE65*	heterozygous	c.11+5G>A; 725+2T>A	[splice];[splice]	39	10.5	No	14.6	12	59	No	No
403	21/F	*RPE65*	heterozygous	c.11+5G>A;c.1102T>C	[splice]; [p.Tyr368His]	23	50	No	8.0	25	26	62%	No
501	40/M	*RPE65*	homozygous	c.495+1 insG	p.Gln165fsX2	11	3	No	No	126	74	No	59%
502	24/F	*RPE65*	heterozygous	c.118G>A; c.272G>A	p.Gly40Ser; p.Arg 91Gln	33	29.5	No	No	145	35	38%	54%
503	26/F	*RPE65*	heterozygous	c.1444delGAT; c.250A>T	p.Asp482del; p.Ile84Phe	3	45	6.0	No	191	135	No	No
601	21/M	*RPE65*	heterozygous	c.986G>A; c.1102T>C	p.Cys329Tyr; p.Tyr368His	8	16.5	No	9.3	100	174	81%	51%
701	23/M	*RPE65*	heterozygous	c.1067insT; c.1178C>G	p.Asn356LysfsX8; p.Ala393Glu	17	61.5	11.7	No	68	54	No	No

^a^ In this study, we defined visual acuity (VA) response as improvement in VA ≥0·1 logMAR (≥5 letter score, or from 0 to seeing any letters) on at least 2 consecutive visits starting by Month 2. The VA response is the mean VA change from baseline across the responding visits.

^b^ GVF response was defined as improvements in GVF retinal area ≥20% on at least 2 consecutive visits starting by Month 2. The GVF response is the mean percent change from baseline in retinal area across the responding visits.

c Patient 401 was considered a responder because she had no measurable VA at baseline but did have measurable VA after treatment.

Ocular examination included best-corrected visual acuity (ETDRS chart), intraocular pressure measurement, electroretinogram, Goldmann kinetic visual field testing, dilated fundus examination and retinal imaging and was conducted before and after treatment. Because this was a proof-of-concept study, there was no pre-specification of primary and secondary outcome measures. In addition, two patients at McGill University were evaluated twice before and twice following drug administration for changes in visual cortex activities, using blood oxygen level detection (BOLD) measured by functional magnetic resonance imaging (fMRI) of the brain (S4 Text). In short, we used three different patterned moving stimuli varying from high to low luminance contrast.

Goldmann visual fields (GVFs) were digitized and converted to functional retinal area in mm^2^ according to a standard protocol (described in S1 Table) [24,25]. For each eye of each patient, a primary isopter (V4e, IV4e, II4e, II4e, or I4e) was chosen where the retinal area fell within the range of 0.7–2.4 log mm² (5–250 mm², equivalent to a circular visual field diameter of 7°–70°). Two visual field assessments were performed prior to treatment, with the average used as baseline.

Full-field electroretinography was recorded according to the International Society for Clinical Electrophysiology of Vision (ISCEV) guidelines [26]. Nine patients showed no response to 31 Hz flicker at baseline or subsequent visits. ERG datasets included a full set (all ISCEV parameters, all visits) for 2 patients, reduced set (all parameters but not at all visits) for 2 patients, and 5 patients with limited parameters at a few visits.

SD-OCT images were collected at all seven participating sites of which six used Spectralis (Heidelberg Engineering) and one used Cirrus OCT (Carl Zeiss Meditec). A high number of frames was averaged in order to improve image quality. In case of nystagmus, volume scan was used to ensure the successful capture of the foveal scan. A custom-designed OCT segmentation program built in IGOR Pro (IGOR Pro 6.12; WaveMetrics, Inc, Lake Oswego, Oregon), similar to a previous technique [20], was used to profile and measure the length of the total retina and individual retinal layers [27]. Average length of the outer segment (OS) layer (measured from the outer segment/retinal pigment epithelium border to the inner segment ellipsoid band) in the central 20° (10° radius around the fovea) was calculated.

Safety evaluations included monitoring of vital signs, electrocardiogram (ECG), heart rate and blood pressure, a battery of clinical laboratory tests including 12 hour fasting serum chemistry, hematology, serum retinol, coagulation testing, thyroid function testing, and urinalysis. Follow-up visits were planned to continue at least through 12 months post treatment (approximately days 7/8, 14/15 and 30; and months 2, 4, 6, 8, 10, and 12) (S1 Text).

Statistical analyses were primarily descriptive statistics of either continuous variables (i.e., mean) or categorical variables (i.e., percentage of “responders”). In the case of functional retinal area and visual acuity, a response of ≥20% or ≥40% improvement and a ≥5 or ≥10 ETDRS letter score improvement at two or more consecutive visits within two months of treatment was used, respectively, to define treatment response. These values were chosen because in earlier studies on a similar patient cohort test-retest variability of visual fields was less than 20% [28]; and the typical variability in visual acuity was shown to be in the same order of magnitude (95% coefficient of reliability of 8.5 ETDRS letter score [29] / 7–12 ETDRS letter score [30]). In addition, patients who had no measurable GVF or visual acuity at baseline were considered to have a positive response if they had measurable GVF or visual acuity on two consecutive visits after treatment. Duration of response was the length of time over which patients continued to meet the response criterion. A post-hoc analysis of time to initiation of response (Kaplan-Meier survival distribution function) was also performed. The analysis was performed on the intent-to-treat data set (18 patients), with no imputation performed for missing values and no protocol deviations that led to exclusion from the analysis. All calculations were performed using SAS (version 8.2, Cary, NC). Details of the analysis of the functional MRI scans are described in S4 Text.

Adverse events (AE) were coded using the Medical Dictionary for Regulatory Activities (MedDRA Version 11.0, Chantilly, Virginia, USA, http://www.meddramsso.com). The study was conducted in accordance with the International Conference on Harmonization (ICH) E6: Good Clinical Practice.

## Results

Seventeen of 18 enrolled patients completed the study. All 18 subjects received the intended once-daily oral dose of 40 mg/m^2^ per day QLT091001 for 7 consecutive days. Each patient had at least 2 months of follow-up and 13 patients (72%) had 8 months of follow-up (for at least one of GVF or visual acuity measurement). One patient requested to be withdrawn for personal reasons after 118 days of follow-up (Fig 1, S1 Text). Patients had a mean age of 28.5 years (range 6 to 55 years; Table 1). There were 10 male and 8 female patients; 11 patients were of Caucasian origin, 6 Asian and 1 Hispanic (S1 Table). Thirteen patients (72%) had mutations in the *RPE65* gene and 5 (28%) in the *LRAT* gene (Table 1). At baseline, mean visual acuity was 30 ETDRS letter score (Snellen equivalent of 20/250) and mean visual field for the primary isopter was 1.8 log mm^2^. The study population is representative of the target therapeutic population.

### Visual fields

**Fig 2:**
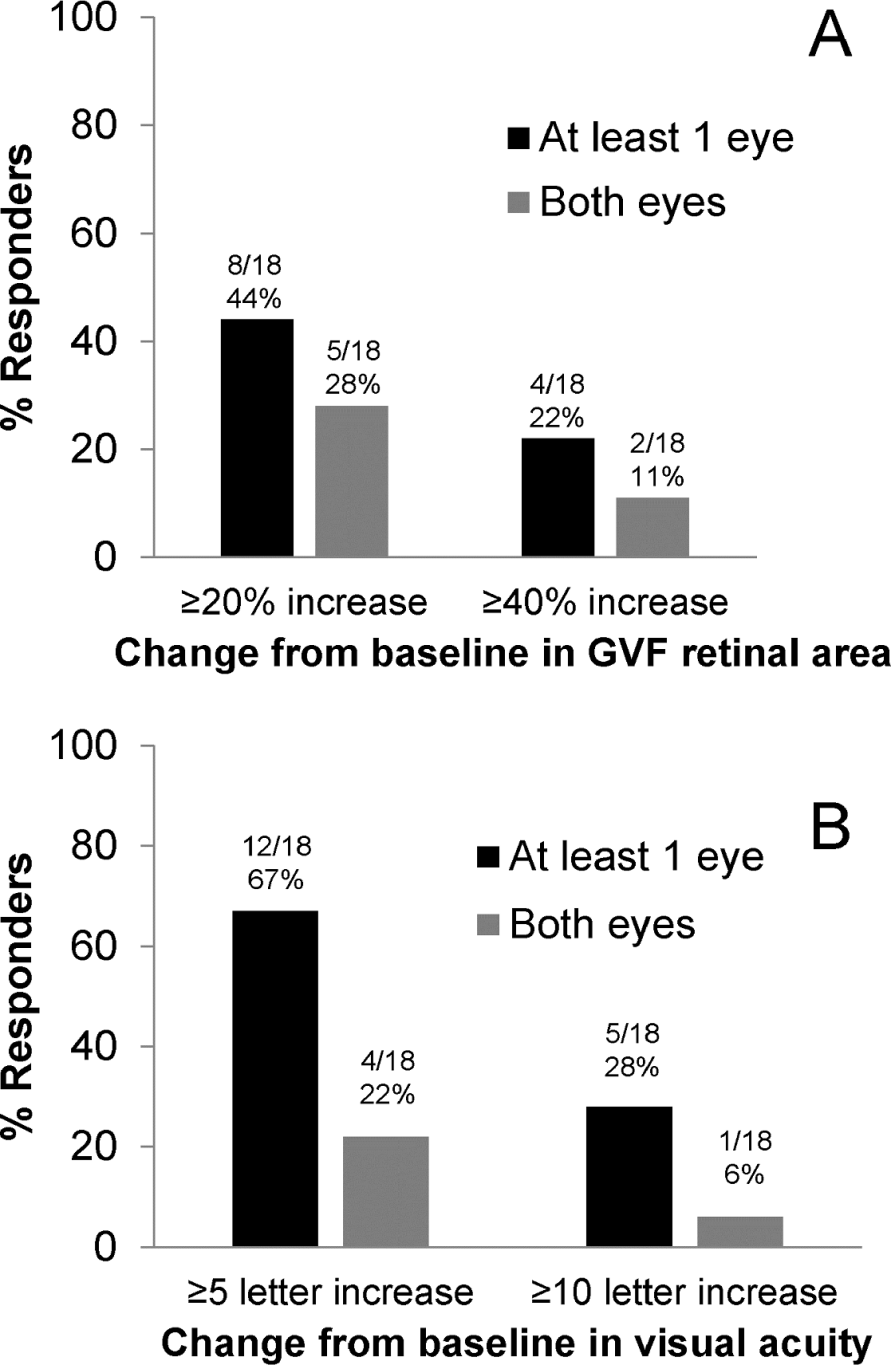
Percent of Treatment Responders for Functional Retinal Area (A, top) and Visual Acuity (B, bottom). Response in functional retina area was defined as an increase in visual field area from baseline of ≥20% increase in the visual field area in the primary isopter in one or both eyes at two or more visits within 2 months of treatment. Visual acuity response was defined as an increase from baseline in visual acuity of ≥5 ETDRS letter score at two or more visits within 2 months of treatment.

**Fig 3:**
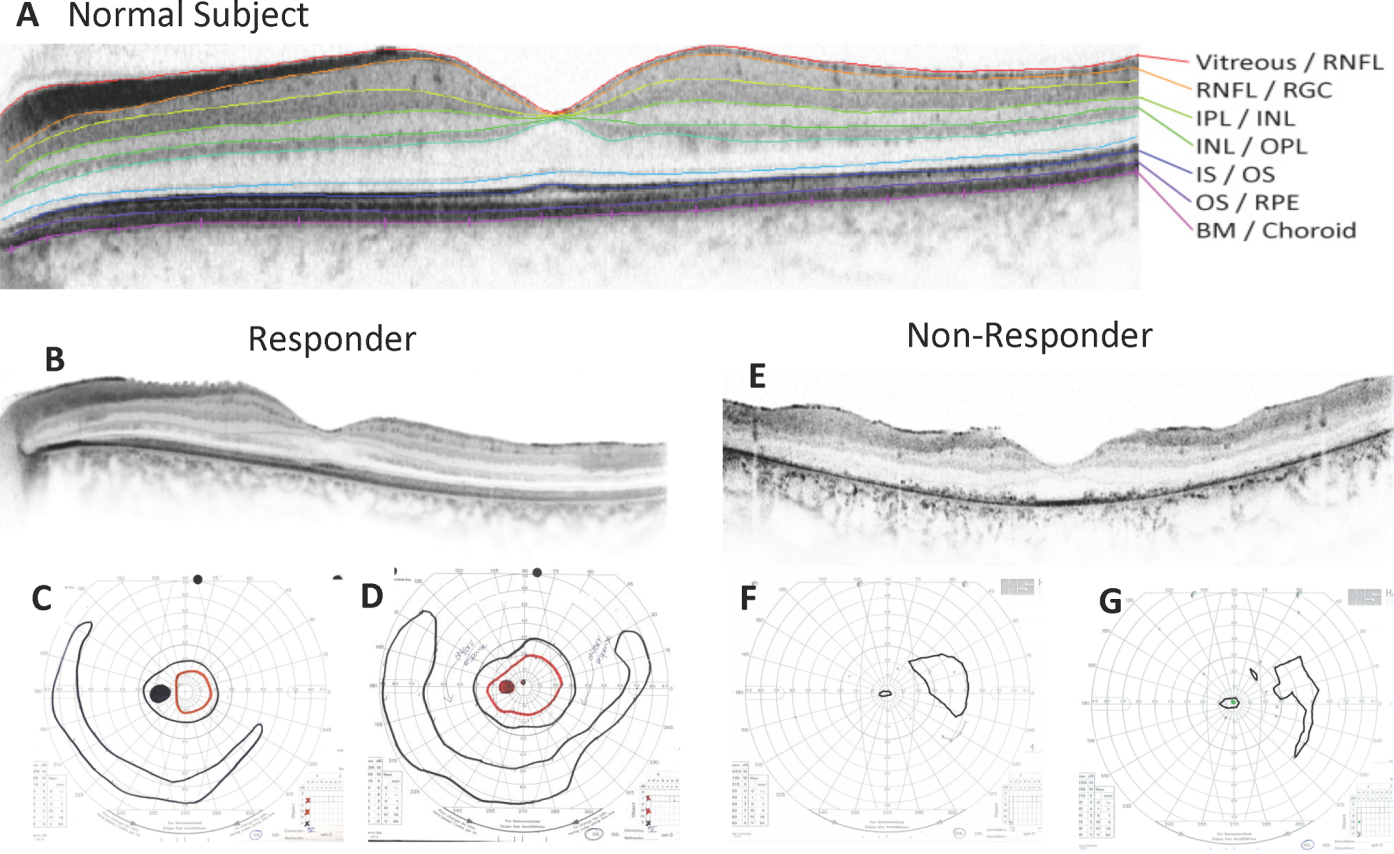
Goldmann Visual Fields and Respective SD-OCT scans of the Central Retina in a Treatment Responder and a Non-Responder. (A) Normal subject. The horizontal midline scan shows segmentation lines separating Vitreous/Retinal Nerve Fiber Layer (RNFL), RNFL/Retinal Ganglion Cell Layer (RGC), Inner Plexiform Layer (IPL)/Inner Nuclear Layer (INL), INL/Outer Plexiform Layer (OPL), Inner segment (IS)/Outer segment (OS), OS/ Retinal Pigment Epithelium (RPE), Bruch’s Membrane (BM)/Choroid. The OS layer lies between the IS/OS line and the OS/RPE line (see Hood et al. 2009 Ref 19). (B) SD-OCT foveal scan at screening (length of OS layer in the central 20° was 23.1 μm), (C) Goldmann Visual Field (GVF) at screening visit (retinal area of primary isopter = 26 mm^2^) and at month 1.5 (D) (retinal area of primary isopter = 81 mm^2^) in a RP patient who showed a treatment response (subject 110 OS, see Table 1). (E) SD-OCT foveal scan at screening (length of OS layer in the central 20° was 6.2 μm), (F) Goldmann Visual Field (GVF) at screening visit (retinal area of primary isopter = 55 mm^2^) and at month 1 (G) (retinal area of primary isopter was 44 mm^2^) of a non-responder (subject 402 OS, see Table 1).

Within 2 months of treatment, 8 of 18 trial participants (44%) showed a ≥20% increase of the functional retinal area in the primary isopter in one or both eyes, and 4 of 18 (22%) showed a ≥40% increase in one or both eyes at two or more consecutive visits within 2 months of treatment (Fig 2A; Tables 1 and 2; see example in Fig 3). The mean duration of this response was approximately 11 weeks (Table 3, S2 Fig). Across all eyes and visits, the mean percent change from baseline in retinal area ranged between -13% and 65% (S1 Fig).

**Table 2:** Functional Retinal Area Responders for Primary Isopter (top) and Visual Acuity Responders (bottom).

		*Number (%) of Patients*							
		*Responder Category (% Change in Retinal Area from Baseline)*							
		*≥20% GVF Increase* ^*a*^				*≥40% GVF Increase* ^a^			
	*N*	*At least 1 eye*		*Both Eyes*		*At least 1 eye*		*Both Eyes*	
LRAT	5	2	(40%)	2	(40%)	1	(20%)	1	(20%)
RPE65	13	6	(46%)	3	(23%)	3	(23%)	1	(8%)
		*Responder Category (VA Change from Baseline)*							
		*≥5 Letter Score Increase* ^*a*^				*≥10 Letter Score Increase* ^*a*^			
	*N*	*At least 1 eye*		*Both Eyes*		*At least 1 eye*		*Both Eyes*	
LRAT	5	4	(80%)	2	(40%)	2	(40%)	0	
RPE65	13	8	(62%)	2	(15%)	3	(23%)	1	(8%)

^a^ For at least 2 consecutive visits starting within 2 months from the start of QLT091001 treatment.

Note: The responder categories include one patient who was not able to read any letters on the ETDRS chart at baseline but who was on-chart post-treatment (both eyes of patient 401 with RPE65 deficiency); inclusion criteria required either best corrected visual acuity (BCVA) of at least 3 letter score or evidence from OCT/FAF of a viable photoreceptor layer.

**Table 3:** Duration of Functional Retinal Area Response for Primary Isopter (top) and Visual Acuity Response (bottom).

		*Duration of GVF Response in Days*					
		*≥20% GVF Increase* ^*a*^			*≥40% GVF Increase* ^a^		
	*N*	*n*	*Mean*	*Min-Max*	*n*	*Mean*	*Min-Max*
LRAT eyes	10	4	123	22–253	2	104	34–174
RPE65 eyes	26	9	49	7–107	4	67	16–107
		*Duration of VA Response in Days*					
		*≥5 Letter Score Increase* ^*a*^			*≥10 Letter Score Increase* ^*a*^		
	*N*	*n*	*Mean*	*Min-Max*	*n*	*Mean*	*Min-Max*
LRAT eyes	10	6	194	47–246	2	129	125–132
RPE65 eyes	26	10	84	13–226	4	104	13–206

^a^ For at least 2 consecutive visits starting within 2 months from the start of QLT091001 treatment.

### Visual acuity

Within 2 months of treatment, 12 of 18 patients (67%) showed a ≥5 ETDRS letter score increase in one or both eyes, and 5 of 18 (28%) showed a ≥10 letter score increase at two or more visits within 2 months of treatment (Fig 2B; Tables 1 and 2). The median time required to obtain the response criterion of a ≥5 letter score increase in visual acuity was 8 days. The mean duration of response was approximately 4 months (Table 3, S4 Fig). The mean change from baseline in visual acuity letter score was determined for each follow-up visit and ranged from 3 to 5 letters (S3 Fig).

### Photoreceptor outer segment length and its relationship to treatment response

The average length of a healthy normal outer segment (OS) is approximately 32 μm [19]. The average baseline length of the OS layer (central 20°) for treatment responders for the visual field (as defined above) was 11.7 μm (64% less than the normal average) and for non-responders 3.5 μm (89% less than normal average; Fig 3). This difference was statistically significant (P = 0.02, Wilcoxon Rank test). Using the Receiver Operating Characteristic (ROC) curve to determine a critical OS length value of the central 20 degrees of the retina that would separate responders from non-responders, an OS length of 7 μm or above was found to best predict a treatment response (χ2 = 17.43; df = 1; p<0.005) [27].

### Functional MRI studies of the cerebral cortex

**Fig 4:**
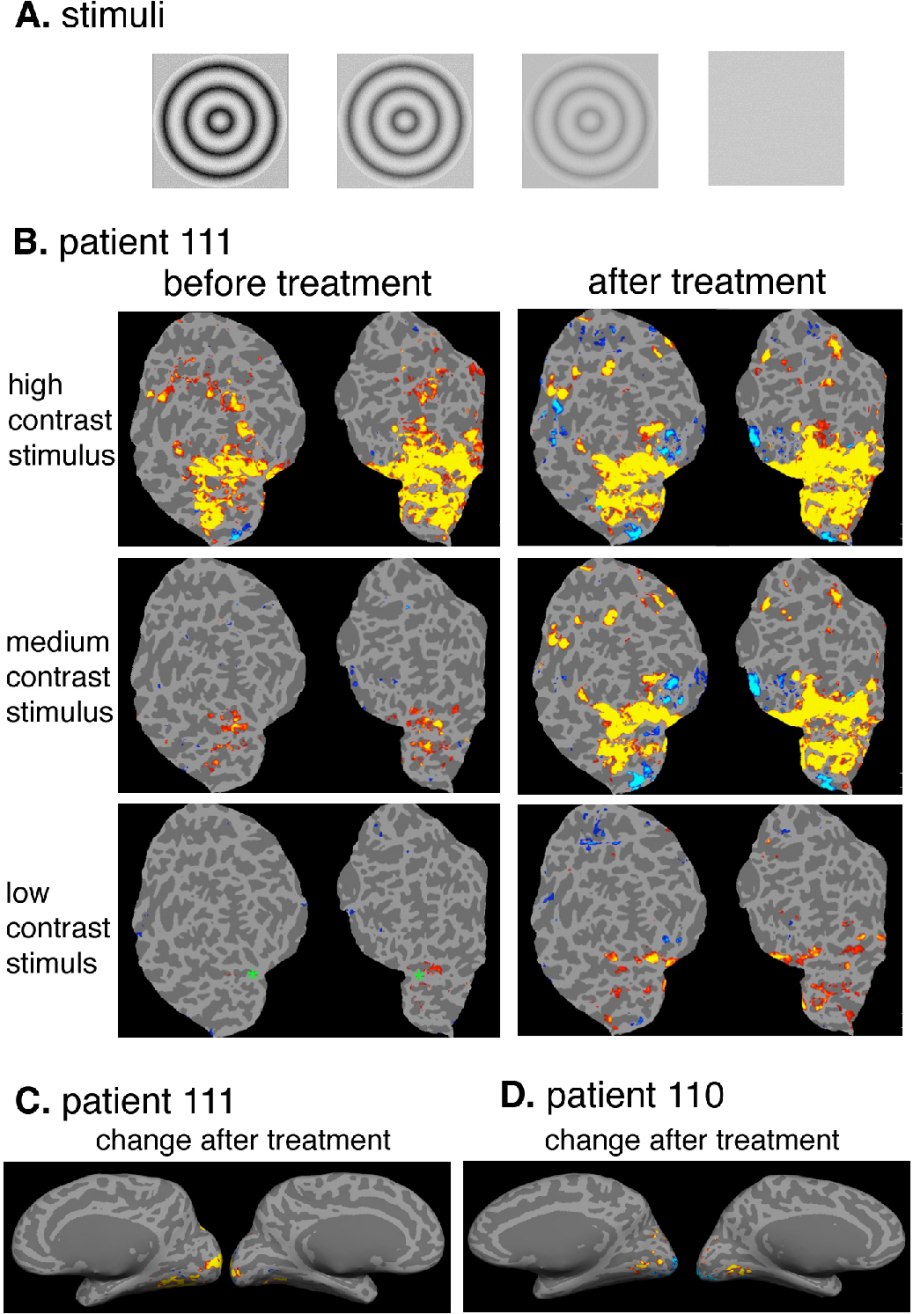
Functional MRI for Two Patients (110 and 111) in Response to Patterned Moving Stimuli Varying from High to Low Luminance Contrast. (A) Depiction of the stimuli at three contrasts, high to low from left to right. (B) For patient 111, before treatment, the visual cortical response was robust for high contrast, modest for medium contrast, and negligible for low contrast. After treatment, the response to medium and low contrast stimuli increased noticeably. The statistical maps for the first (before treatment) and the fourth (after treatment) session are shown (0.01 < p < 0.0001, FDR), each on a flattened representation of the posterior cortex, for both left and right hemisphere. The occipital pole is marked in the lower left panel with a green asterisk for orientation; v-shaped margin of cortex represents the split calcarine sulcus. (C) For patient 111, for medium contrast stimulus, the regions in medial occipital cortex that show significant *increased response* for all post-treatment scans compared to all pre-treatment scans are shown on medial view inflated brain. (D) Similar results were also obtained for patient 110.

Two patients (110 and 111) at one site (McGill University) were available to complete a functional MRI assessment. Testing was attempted for a third patient (117), but at age 6 he was too young to provide meaningful data. For patient 110, scans occurred 7 days and 5 days prior to the start of treatment, and 9 and 13 days after. For patient 111, scans occurred 4 days and 2 days prior to the start of treatment, and 10 and 11 days after. BOLD (blood oxygen level-dependent) scans (256 seconds each) were done in a 3T Siemens TRIO scanner with a voxel resolution of 4 mm x 4 mm x 4 mm. Patients viewed moving, patterned stimuli at one of three luminance contrasts (Fig 4A) and a homogeneous mid-level gray screen with a fixation mark only. Fig 4 shows that visual cortical activation increased after treatment in both patients. Moreover, the cortical response increased systematically as stimulus contrast was increased, as would be predicted for normal subjects, and strongly suggests a visually driven change. The statistical maps for patient 110 for the first (before treatment) and the fourth (after treatment) session are shown (0.01 < p < 0.0001, false discovery rate [FDR]), each on a flattened representation of the posterior cortex, for both left and right hemisphere. Regions that showed significant increase after treatment with a repeated-measures statistical design (high and medium contrast stimuli) were located primarily in medial occipital cortex in both patients, presumably overlapping with primary visual cortex (V1) (Fig 4C and 4D). In addition, patient 111 also had a bilateral region of BOLD increase in lateral occipital cortex (posterior middle temporal sulcus), close to a region in normal subjects known to have high sensitivity to visual motion called MT+ [31]. We repeated the fMRI testing on both patients again, for a second time after dosing and found the same results, the medium contrast stimuli significantly increased both in the extent and intensity of the cortical response in both the left and right hemisphere of the occipital cortex (Fig 4).

### Safety and other clinical measures

Nine of 18 (50%) patients had detectable cone electroretinogram (ERG) signals to 31 Hz flicker both pre- and post-treatment, with amplitudes that were not significantly affected by the treatment. There was no treatment related gain in ERG responses observed; also, for subjects who did not show responses pre-treatment there was no gain of detectable responses after treatment (for an example see S5 Fig). There were no deaths, withdrawals due to adverse events (AE), or serious adverse events. All patients enrolled in the study experienced at least one AE that was related to treatment. A total of 209 AEs were reported, with 141 of these events considered by the investigator to be related to treatment. The most common treatment-related AE was headache, occurring in 94% of patients (17/18). Other common treatment-related AEs included increased serum aspartate aminotransferase (33%), photophobia (28%), alanine aminotransferase (22%), nausea (17%) and reduced high density lipoprotein (17%) (For a summary of all adverse drug reactions see S2 Table).

One patient (an 11-year old male) experienced an unexplained 13 ETDRS letter score decrease in visual acuity during the screening period in the left eye and a further loss of 12 letter score after treatment until Month 1. The vision subsequently improved and by Month 6 there was a decrease of 3 letter score compared to baseline. The same patient experienced photopsia in both eyes on Days 2, 3 and 4, and photophobia in both eyes starting on Day 92 that was still present when the patient completed the study. The cause of the visual acuity decrease is not known. The patient’s fundus appearance, OCT and full-field ERG amplitude measures were not affected.

Treatment with QLT091001 resulted in short term deviations from normal in a number of laboratory parameters including decreased hematocrit, decreased white blood cell count, increased alanine aminotransferase, increased aspartate aminotransferase, increased cholesterol, increased low density lipoprotein, increased triglycerides, decreased high density lipoprotein, increased bicarbonate, increased potassium, decreased total protein and decreased free thyroxine. Most of these parameters had returned to normal within 1 month; cholesterol and triglyceride levels returned to normal by Month 2 and hematocrit by Month 4. There were no clinically significant changes in other clinical measures (biomicroscopy, dilated fundus exam, IOP, ECG, vital signs, subjective refraction or cycloplegic refraction).

## Discussion

Restoring vision in retinal degenerative disease is an unmet medical need. In *RPE65*- and *LRAT*-related human RP, there is significant photoreceptor dysfunction and retinal degeneration that ultimately results in legal blindness. The positive results obtained in *rpe65-/-*mice using 9-*cis*-retinyl acetate clearly warranted the development of a similar therapeutic approach for humans. A number of novel therapies such as gene replacement therapy [32–34] (summarized in [35]) and biological growth factors [36,37] (summarized in [38]) are currently being tested in human clinical trials. Visual restoration has been achieved in transgenic mice and naturally occurring mouse and dog retinal degeneration models by treating these animals with both subretinal gene replacement therapy as well as pharmacologically with 9-*cis*-retinyl acetate [16].

This study showed that one week of daily 9-*cis*-retinyl acetate treatment can improve visual function in some subjects with RPE65 and LRAT deficiency. We cannot rule out that a placebo effect may have contributed to the improved measures of vision but it would be very unusual to see improvements of retinal function of this magnitude in progressive retinal disease. Another factor that may have influenced the results is the variability in the responses of visual field testing. In a previous study in patients with RP and a visual field radius greater than seven degrees, test-retest variability was less than 20% [28], but in another study on patients with LCA carrying mutations in *RPE65*, inter-visit variability was larger [29]. In the current study we attempted to minimize variability firstly by standardization of test conditions across all participating centers and training and accreditation of those undertaking the visual field tests before the start of the study [39]. We also minimized variability through the choice of the primary isopter (reducing inter-individual variability of functional retinal area to only one log unit) and by evaluating all visual fields at a central reading center. The requirement to meet the treatment response criterion for at least two consecutive visits within two months of treatment provided additional assurance that the improvement in visual field represented a treatment effect. Similar considerations apply to the visual acuity measurements obtained in the trial.

The correlation between retinal structure and function (response to treatment) indicates a statistically significant association between baseline OS length and treatment response (as measured with GVF) to QLT091001. A remaining OS layer of 7 μm or more appears to allow at least some restoration of phototransduction within photoreceptors with the synthetic 9-*cis*-retinyl acetate and thus to mediate the first step in the visual cascade. The improvement of visual fields suggests that the bipolar cells and proximal visual system still effectively receive synaptic inputs from the surviving photoreceptors.

Deficiency in RPE65 and LRAT causes a spectrum of retinal disease that is typically classified as LCA (LCA2; OMIM #204100) or RP (RP20; OMIM 613794#) [1], but it may include other terms such as early-onset severe rod-cone dystrophy [40]. The difference in phenotypic classification may explain why in an earlier trial, in which an independent cohort of patients with the diagnosis of LCA were enrolled in a single center, improvements in visual function were overall larger [18]. Also, the cohort of LCA patients was significantly younger and therefore may have exhibited less degeneration to date, which may have allowed more significant visual restoration. In fact, 16 of 28 LCA eyes (57%) had outer segment length in the central 20 degrees >7 microns, compared to only 10 of 36 RP eyes (27%). The type of genetic defect (*LRAT* or *RPE65*) did not appear to affect how LCA patients responded to the drug [18] and this was also true for RP patients (Table 2). While improvements on psychophysical tests were observed, there were no detectable improvements observed on standard electroretinography, similar to what has been previously observed in the gene therapy trials [33]. The lack of treatment related electrophysiological improvement suggests that the retinal functional improvements even in the most responsive patients are still below the threshold of the standard ERG. This is consistent with the relative severity of retinal degenerations due to mutations in *RPE65* or *LRAT*. It was recently suggested that, once the disease has progressed to the point that no amplitudes are recordable by electroretinography, visual field measurements are the best parameters left to monitor such disease [41]. The current activities to treat *RPE65*-related retinal dysfunction with gene therapy and pharmacotherapy appear to be complementary. While the current gene therapies use subretinal delivery, a limited area of the retina in one eye at the time is being treated, while treatment with QLT091001 is a systemic treatment with treatment effects on presumably all remaining photoreceptors in a given retina, in both eyes. It is conceivable that patients that were previously treated with gene therapy could have an additional benefit when being treated with QLT091001, but such a trial has not been done to date, and thus this possibility remains speculative.

The observations in visual function measurements of this study are consistent with pharmacokinetic (PK) findings in single dose and radiolabelled single-dose PK studies with QLT091001 (Data on File, QLT Inc.) and also the observation that some agents in the oral retinoid class, particularly those with high lipophilic properties show longer half-lives and lengthy persistence [42]. The safety profile of QLT091001 in this study is consistent with that observed for other retinoid compounds, with patients experiencing adverse events such as headache and facial flushing. Oral QLT091001 of doses from 1.25-60 mg/m^2^ has been evaluated in a series of safety studies in normal healthy volunteers. While adverse effects such as headaches, nausea and cutaneous flushing were seen at the highest doses they were transient (Data on File, QLT Inc). Headache was noted in a dose–related fashion with essentially no headaches noted below the 20 mg/m^2^ dose in healthy volunteer studies (Data on File, QLT Inc). Photophobia was also common in both healthy volunteers and IRD patients and is not a typical adverse reaction of other retinoids. Possibly, this may be related to improved function of photoreceptors.

In summary, the results of this open-label proof-of-concept study using one 7-day oral dose of QLT091001 demonstrate an acceptable safety profile and an improvement in the visual field and/or visual acuity in a majority of patients with RP due to mutations in *RPE65* and *LRAT*. A subset of patients who underwent fMRI showed an increase in visual cortical activation after treatment. OCT measurement of outer segment length may predict treatment response.

## Supporting Information

S1 TREND Checklist TREND Statement Checklist. (PDF)

S1 Fig Scatterplots Showing Individual Functional Retinal Area (GVF Retinal Area of Primary Isopter) at Every Visit for All Eyes Against Baseline. (PDF)

S2 Fig Onset and Duration of Visual Field Response in Responding Eyes by Patient. (PDF)

S3 Fig Scatterplots Showing Individual ETDRS Letter Score at Every Visit for All Eyes Against Baseline. (PDF)

S4 Fig Onset and Duration of Visual Acuity Response in Responding Eyes by Patient. (PDF)

S5 Fig Full-Field Standard Electroretinography (ERG): Original Tracings and Fourier Analysis of the 31-Hz Photopic Flicker ERG of Subject #601 at Baseline and Post-Treatment (2 months). (PDF)

S1 Protocol Study Protocol. (PDF)

S1 Table Demographics. (PDF)

S2 Table Summary of All Adverse Drug Reactions. (PDF)

S1 Text Visit Schedule. (PDF)

S2 Text Exclusion Criteria. (PDF)

S3 Text Calculation of Functional Retinal Area. (PDF)

S4 Text Details for Functional Magnetic Resonance Imaging. (PDF)

S5 Text List of Participating Sites. (PDF)
