# Supplementary material for: Safety and Proof-of-Concept Study of Oral QLT091001 in Retinitis Pigmentosa Due to Inherited Deficiencies of Retinal Pigment Epithelial 65 Protein (RPE65) or Lecithin:Retinol Acyltransferase (LRAT)
Source: PLoS One. 2015 Dec 10;10(12):e0143846. doi: 10.1371/journal.pone.0143846 (PMC4687523; doi:10.1371/journal.pone.0143846)

S1 Fig. Scatterplots Showing Individual Functional Retinal Area (GVF Retinal Area of Primary Isopter) at Every Visit for All Eyes Against Baseline. The dotted line shows no improvement and the dashed line the threshold of a 20% improvement in functional retinal area.

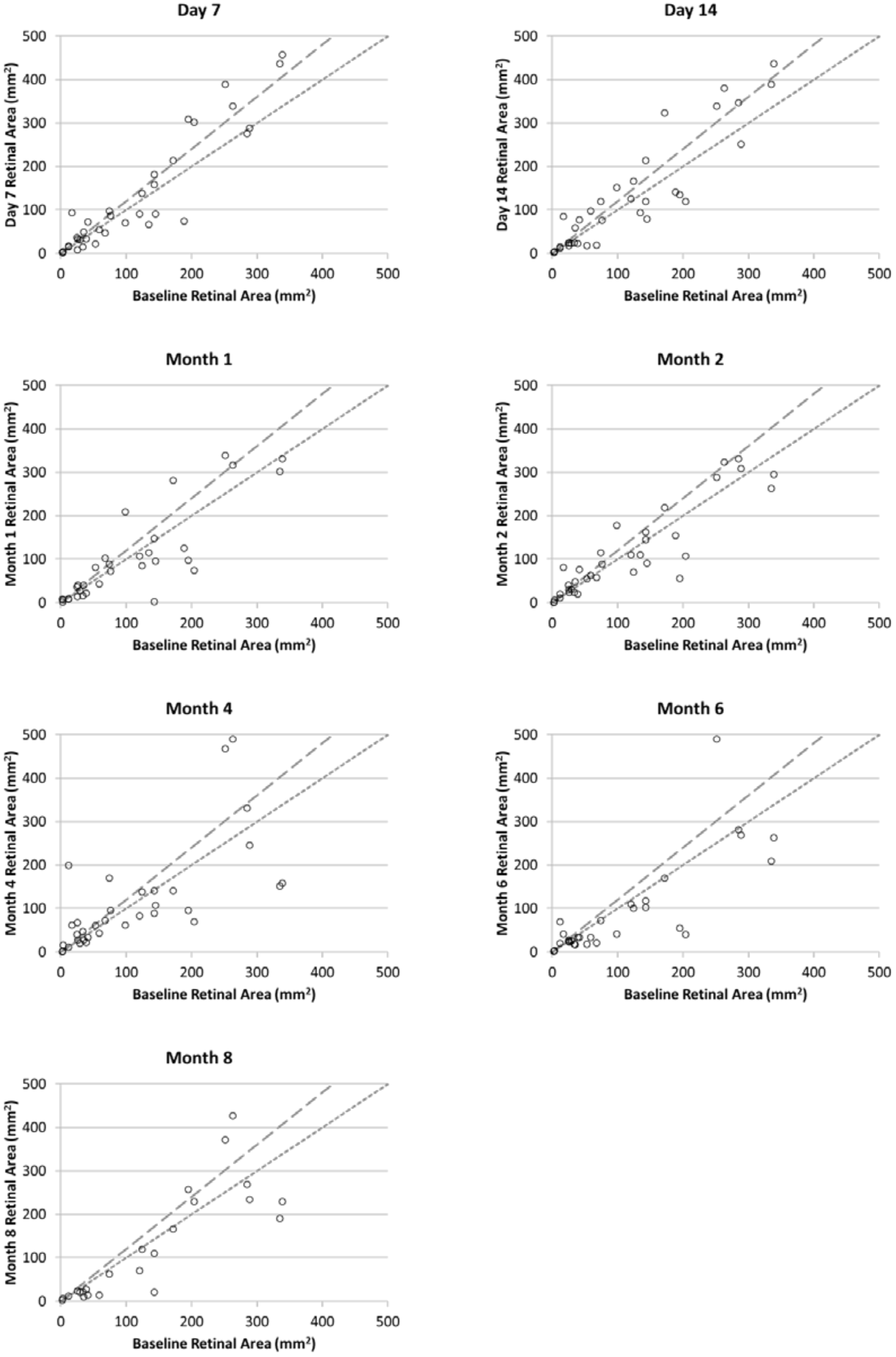

Supplement: S1 Fig — (PDF) [file pone.0143846.s002.pdf]
