# Supplementary material for: Safety and Proof-of-Concept Study of Oral QLT091001 in Retinitis Pigmentosa Due to Inherited Deficiencies of Retinal Pigment Epithelial 65 Protein (RPE65) or Lecithin:Retinol Acyltransferase (LRAT)
Source: PLoS One. 2015 Dec 10;10(12):e0143846. doi: 10.1371/journal.pone.0143846 (PMC4687523; doi:10.1371/journal.pone.0143846)

S2 Fig. Onset and Duration of Visual Field Response in Responding Eyes by Patient. Response was defined as an increase in functional retinal area for the primary isopter from baseline of ≥20% at two or more consecutive visits within 2 months of treatment. Symbols represent time points at which visual field assessments were performed. Duration of response (shown by the horizontal lines) is the period over which the patient's functional retinal area remained above the response criterion of a ≥20% increase.

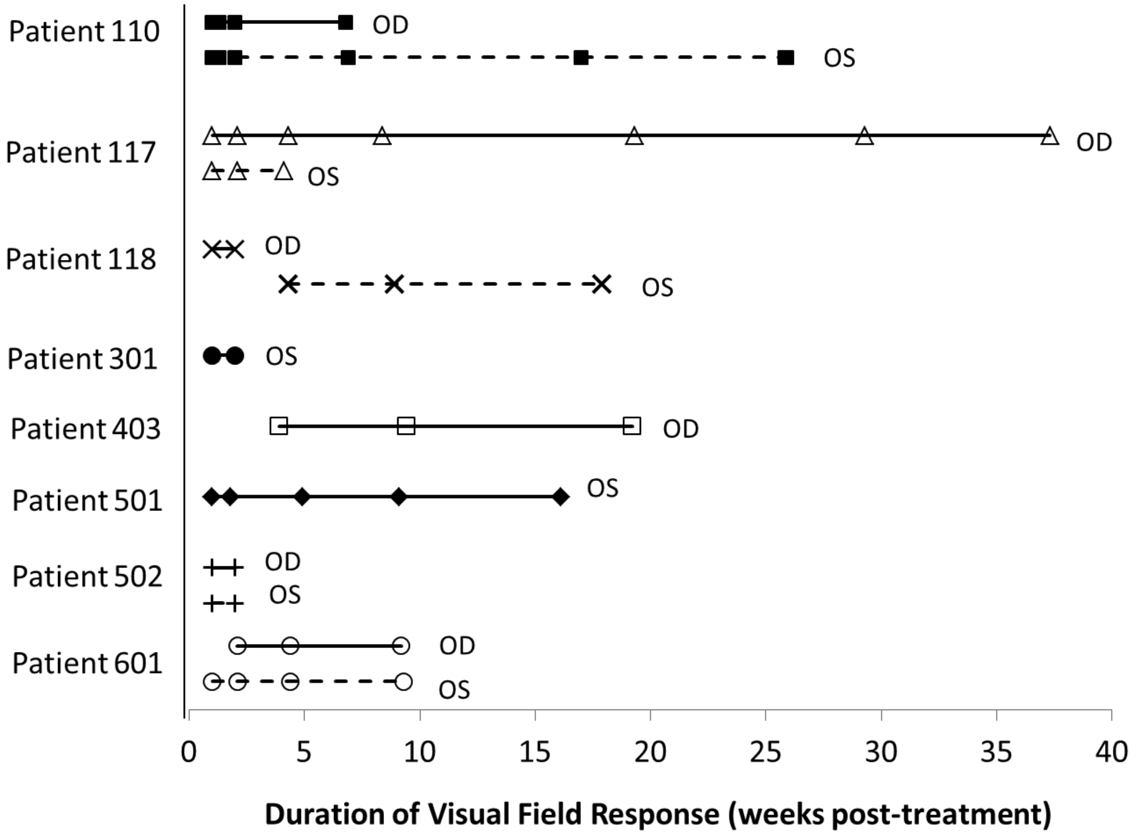

Supplement: S2 Fig — (PDF) [file pone.0143846.s003.pdf]
