# Supplementary material for: Safety and Proof-of-Concept Study of Oral QLT091001 in Retinitis Pigmentosa Due to Inherited Deficiencies of Retinal Pigment Epithelial 65 Protein (RPE65) or Lecithin:Retinol Acyltransferase (LRAT)
Source: PLoS One. 2015 Dec 10;10(12):e0143846. doi: 10.1371/journal.pone.0143846 (PMC4687523; doi:10.1371/journal.pone.0143846)

S3 Fig. Scatterplots Showing Individual ETDRS Letter Score at Every Visit for All Eyes Against Baseline. The dotted line shows no improvement and the dashed line the threshold of a 5 letter score improvement.

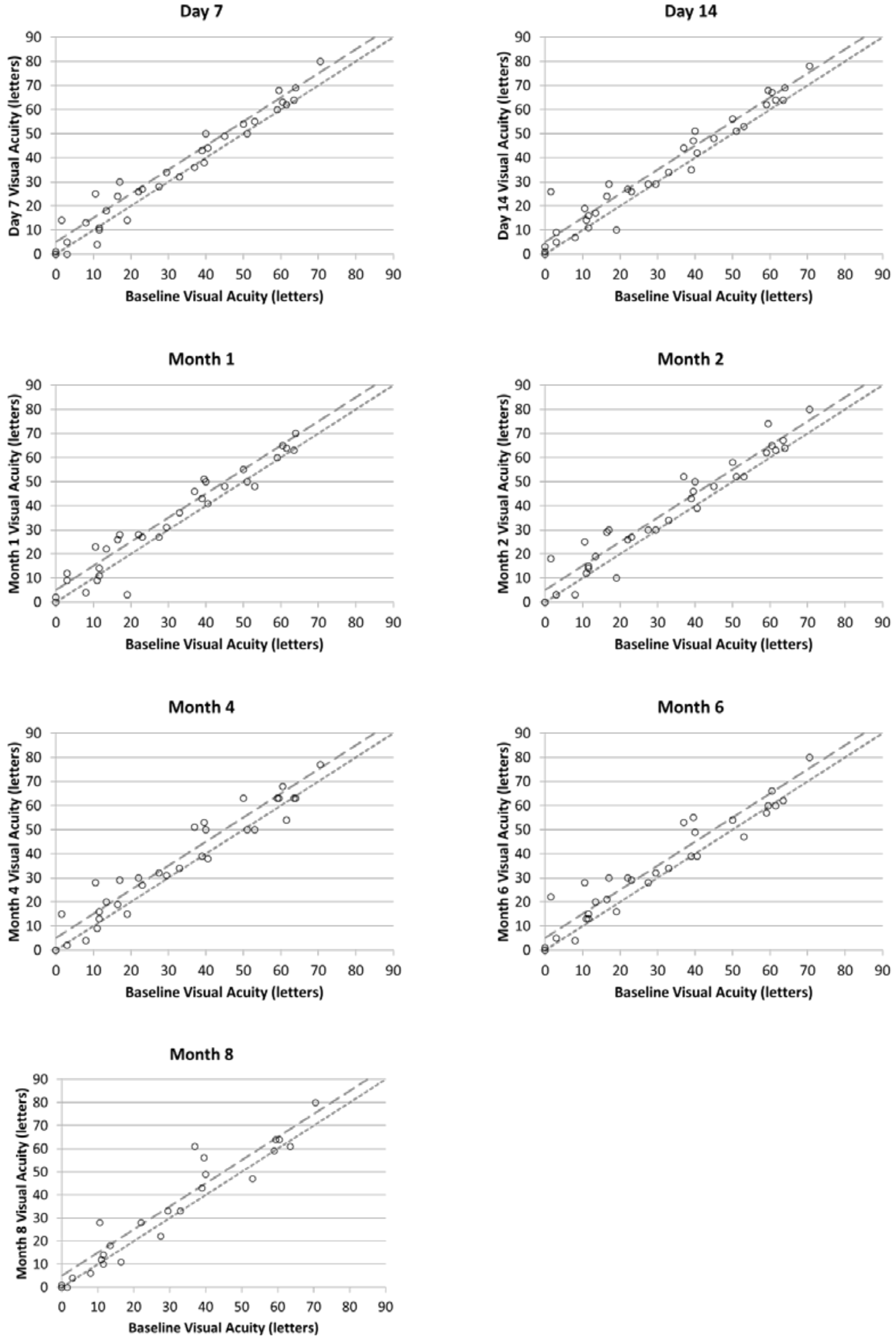

Supplement: S3 Fig — (PDF) [file pone.0143846.s004.pdf]
