# Supplementary material for: Safety and Proof-of-Concept Study of Oral QLT091001 in Retinitis Pigmentosa Due to Inherited Deficiencies of Retinal Pigment Epithelial 65 Protein (RPE65) or Lecithin:Retinol Acyltransferase (LRAT)
Source: PLoS One. 2015 Dec 10;10(12):e0143846. doi: 10.1371/journal.pone.0143846 (PMC4687523; doi:10.1371/journal.pone.0143846)
Supplement: S5 Fig — (PDF) [file pone.0143846.s006.pdf]

S5 Fig. Full-Field Standard Electroretinography (ERG): Original Tracings and Fourier Analysis of the 31-Hz Photopic Flicker ERG of Subject #601 at Baseline and Post-Treatment (2 months). Patient was a 21 year-old male affecting by retinitis pigmentosa being compound heterozygous for mutations in RPE65 (see Table 1). The left panels show the original tracings for the right eye (red) and left eye (blue) for a full-field stimulus of a 31-Hz white flicker (ISCEV standard flash). The right upper and middle panels show a Fourier analysis of the responses of the right and left eye, respectively. The lower panels show the ERG response amplitude of the first harmonic (at 31 Hz) together with the noise level (threshold). The responses at baseline are below the noise level. The ERG responses post-treatment at two months are unchanged in amplitude and suggest that the results are above the noise level. Overall, there was no apparent improvement in ERG response after treatment.

Reference: Strasser T, Peters T, Jägle H, Zrenner E, Wilke R (2010) An integrated domain specific language for post-processing and visualizing electrophysiological signals in Java. Conf Proc IEEE Eng Med Biol Soc; 2010: 4687-90.

Baseline (Pre-Treatment):

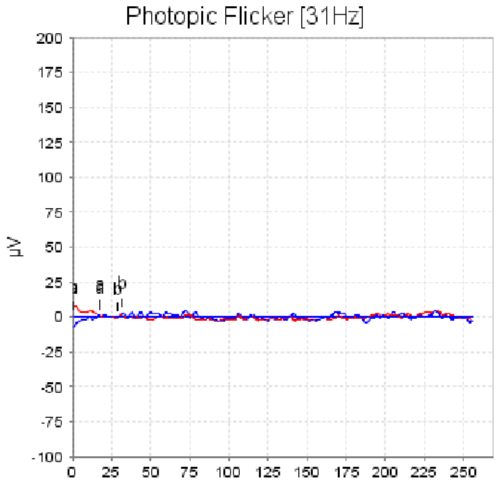

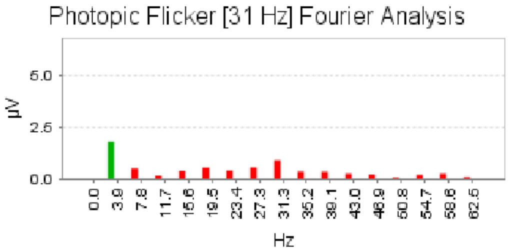

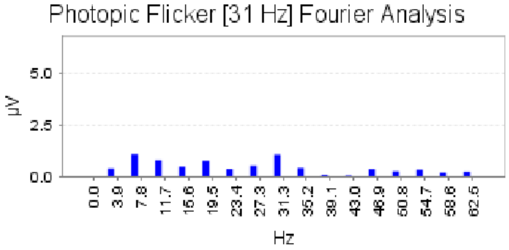

OD	31 Hz Amp_{pp}	1.77 uV
	Threshold	2.43 uV
OS	31 Hz Amp_{pp}	2.08 uV
	Threshold	2.59 uV

Two Months (Post-Treatment):

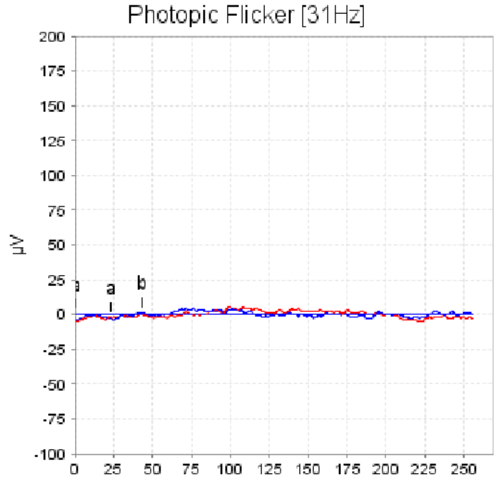

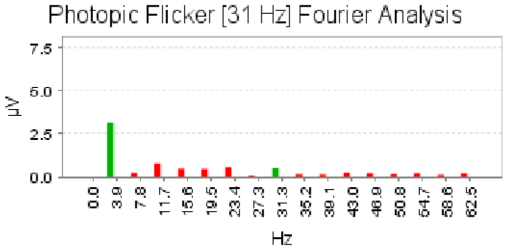

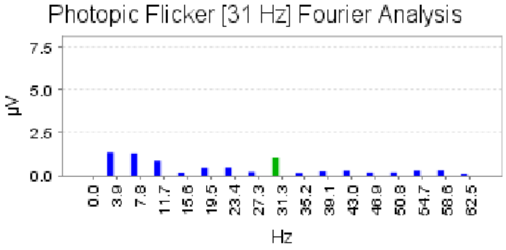

OD	31 Hz Amp_{pp}	1.03 uV
	Threshold	0.65 uV
OS	31 Hz Amp_{pp}	2.06 uV
	Threshold	1.02 uV
