# Supplementary material for: Safety and Proof-of-Concept Study of Oral QLT091001 in Retinitis Pigmentosa Due to Inherited Deficiencies of Retinal Pigment Epithelial 65 Protein (RPE65) or Lecithin:Retinol Acyltransferase (LRAT)
Source: PLoS One. 2015 Dec 10;10(12):e0143846. doi: 10.1371/journal.pone.0143846 (PMC4687523; doi:10.1371/journal.pone.0143846)
Supplement: S1 Protocol — (PDF) [file pone.0143846.s007.pdf]

Clinical Study Protocol — RET IRD 01

QLT091001

An Open-Label, Phase 1b Safety/Proof-of-Concept Study to Evaluate the Effects of Oral QLT091001 in Subjects with Leber Congenital Amaurosis (LCA) or Retinitis Pigmentosa (RP) Due to Inherited Deficiencies of Retinal Pigment Epithelial 65 Protein (RPE65) or Lecithin:Retinol Acyltransferase (LRAT)

Protocol: 11 May 2009
Revised Protocol, Including Amendment 1: 07 July 2009
Revised Protocol, Including Amendment 2: 16 October 2009
Revised Protocol, Including Amendment 3: 27 January 2010
Revised Protocol, Including Amendment 4: 27 April 2010
Revised Protocol, Including Amendment 5: 01 September 2010
Revised Protocol, Including Amendment 6: 15 October 2010
Revised Protocol, Including Amendment 7: 15 March 2011
Revised Protocol, Including Amendment 8: 20 April 2011
Revised Protocol, Including Amendment 8.1 (UK only): 07 July 2011
Revised Protocol, Including Amendment 8.2 (US only): 14 July 2011
Revised Protocol, Including Amendment 8.2.1 (US only): 18 August 2011
Revised Protocol, Including Amendment 8.3 (Germany only): 24 August, 2011
Revised Protocol, Including Amendment 9 (Canada, The Netherlands, UK, and US only): 20 September 2011
Revised Protocol, Including Amendment 9.1 (Germany only): 12 October 2011
Revised Protocol, Including Amendment 10 (All Regions): 19 December 2011

Redacted Version:

Sections of this protocol have been redacted in order to protect the privacy of signatories and vendors, and to protect information that is not relevant to assessing the study and manuscript (such as the preclinical data). Sections describing the study design, patient population, inclusion and exclusion criteria, study procedures, outcome measures, and planned analysis are provided in full.

This document is a confidential communication of QLT Inc. Acceptance of the document constitutes agreement by the recipient that the contents will not be disclosed to any unauthorized personnel, without the prior written authorization of QLT Inc.

QLT Inc.
887 Great Northern Way, Suite 101
Vancouver, British Columbia
Canada V5T 4T5

PROTOCOL APPROVAL

Protocol Number: RET IRD 01

Title of Protocol: An Open-Label, Phase 1b Safety/Proof-of-Concept Study to Evaluate the Effects of Oral QLT091001 in Subjects with Leber Congenital Amaurosis (LCA) or Retinitis Pigmentosa (RP) Due to Inherited Deficiencies of Retinal Pigment Epithelial 65 Protein (RPE65) or Lecithin:Retinol Acyltransferase (LRAT)

< *signatories redacted* >

PROTOCOL SUMMARY

Study Number and Title:<br>RET IRD 01: An Open-Label, Phase 1b Safety/Proof-of-Concept Study to Evaluate the Effects of Oral QLT091001 in Subjects with Leber Congenital Amaurosis (LCA) or Retinitis Pigmentosa (RP) Due to Inherited Deficiencies of Retinal Pigment Epithelial 65 Protein (RPE65) or Lecithin:Retinol Acyltransferase (LRAT)
Clinical Phase: 1
Study Objective:<br>• To evaluate whether 7-day treatment with oral QLT091001 can improve visual function in subjects with Leber congenital amaurosis (LCA) or retinitis pigmentosa (RP) caused by *RPE65* or *LRAT* gene mutations<br>• To evaluate duration of visual function improvement (if observed) in subjects with LCA or RP caused by *RPE65* or *LRAT* gene mutations after 7-day treatment with oral QLT091001<br>• To evaluate the safety of oral QLT091001 administered once daily for 7 days in subjects with LCA or RP caused by *RPE65* or *LRAT* gene mutations
Study Design:<br>This will be an open-label, proof-of-concept, Phase 1b study. The study will include up to 28 subjects with LCA or RP due to inherited deficiency of *RPE65* or *LRAT* (up to 14 subjects for each disease cohort). Subjects in each cohort (including 2 gene mutation subgroups) will receive a once-daily oral loading dose of QLT091001 (40 mg/m^2) for 7 days. This was the highest dose administered in Study RET HV 01. Subjects with either LCA or RP will be enrolled in 1 study center in Canada, a maximum of 5 subjects with RP will be enrolled at approximately 2-3 study centers in the United States, and adult subjects with RP will be enrolled at approximately 3 study centers in the European Union.<br>Subjects will be treated on an outpatient basis but will receive study treatment in the research clinic under medical supervision for each day of treatment. During the study treatment period and for 7 days post-treatment, subjects will be required to limit vigorous physical activity and will be instructed to follow dietary guidelines to avoid excessive vitamin A intake to reduce the influence of such factors on the assessment of safety variables in this study.<br>Each subject will have both eyes evaluated. Follow-up visits will continue through 12 months post-treatment, although subjects will complete the study at the Day 30 visit, or at any visit after that, if they meet entry criteria and are enrolled into a subsequent study for retreatment.<br>To determine proof-of-concept, subjects will undergo visual function testing on both eyes, including: best-corrected visual acuity (BCVA) using Early Treatment Diabetic Retinopathy Study (ETDRS) testing (monocular and binocular tests); color vision testing; visual field testing; and full-field electroretinogram (ERG) to measure cone and rod a- and b-wave amplitudes.
Study Population:<br>The study plans to enroll up to 28 subjects with LCA or RP due to RPE65 deficiency or LRAT deficiency. Subjects with LCA must be 5-65 years of age (inclusive), and subjects with RP must be 18-65 years of age (inclusive) except in the US, where subjects with RP may be 8-65 years of age (inclusive). All subjects must have a best-corrected ETDRS visual acuity of **3 letters** or better **(20/800 Snellen equivalent)**. Subjects who have a lower ETDRS score are still eligible if spectral domain optical coherence tomography (OCT) and fundus autofluorescence (FAF) reveal evidence of a viable photoreceptor layer.
Study Treatment:<br>Subjects will receive a 40 mg/m^2 oral loading dose of QLT091001 once daily for 7 days in the research clinic under medical supervision. The dose of 40 mg/m^2 was the highest dose administered in the dose-escalation safety and tolerability study of healthy adult volunteers (Study RET HV 01; initiated in the middle of 2008 and completed in the first quarter of 2009).

Study Variables:

Preliminary Effect:

- BCVA ETDRS score
- Color vision
- Full-field ERG
- Visual field
- Dynamic pupillometry
- Nystagmus testing
- OCT and FAF
- Subject questionnaire

Other:

- QLT091001 and metabolite concentrations (exploratory)

Safety:

- Vital signs (heart rate, blood pressure, respiratory rate, temperature)
- ECG in triplicate
- Clinical laboratory tests (12-hour fasting serum chemistry and hematology, coagulation testing, thyroid function testing, serum retinol, and urinalysis)
- Biomicroscopic examination
- Intraocular pressure (IOP) and dilated fundus exam
- Height
- Visual acuity
- Adverse events (AEs)
- Concomitant medications

Study Procedures and Assessments:

Screening: Subjects will undergo a Screening period (Day -21 to Day -3) during which all visual function tests and safety assessments, including a serum pregnancy test, will be conducted before starting study treatment. On Day -2/-1, subjects will undergo repeat assessment of visual function tests and other assessments (including another serum pregnancy test) to confirm their eligibility to participate in the study. BCVA ETDRS will be measured twice between Day -2 and Day 0 (for a total of 3 pretreatment measurements, including the Screening measurement).

Treatment: On Day 0, each subject will receive the first dose of study drug. Treatment will be administered for 7 consecutive days (Day 0 to Day 6, inclusive). BCVA tests will be repeated on Day 1. Blood samples for QLT091001 and metabolite analysis will be collected both 4 hours and 24 hours after the first, third, and seventh treatment doses (i.e., 4 hours postdose on Days 0, 2, and 6, and before breakfast on Days 1, 3, and Day 7). Vital signs will be evaluated at Screening, on Day -1, predose and 4 hours postdose on treatment days. AEs and concomitant medications will be monitored at every visit. Triplicate 12-lead ECG recordings and clinical laboratory tests will be performed at Screening, on Day -1, and on Day 3 and Day 7.

Post-treatment: Subjects will have follow-up visits on Days 7/8, 14/15 and 30; and Months 2, 4, 6, 8, 10, and 12. All visual function and safety assessments will be done on Day 7/8 (24/48 hours after taking the last dose of study drug). Visual function tests and safety assessments will be done on Day 14/15 and each subsequent visit. Women and girls of child-bearing potential will have urine pregnancy tests on Day 14/15, Day 30, and Month 2. AEs and concomitant medications will be monitored at every visit.

Sample Size and Statistical Analyses:

The sample size of 28 subjects with LCA or RP due to RPE65 or LRAT deficiency is based on the small population of patients with these diseases and genotypes and on the clinical judgment that this is a sufficient number of subjects to meet the objectives of the study.

- AEs will be coded using the Medical Dictionary for Regulatory Activities (MedDRA) with the number and percentage of subjects experiencing an AE and the total number of events summarized by system organ class and preferred term.
- Concomitant medications will be coded with the World Health Organization Drug Dictionary and tabulated.
- Safety variables including laboratory tests, ophthalmologic examinations and procedures, and vital signs will be summarized with appropriate descriptive statistics by gene mutation subgroup, and disease cohort.
- ECG results, biomicroscopy, IOP, dilated fundus exam, height, and QLT091001 and metabolite levels (if available) will be listed by subject.
- ERG, BCVA scores measured by ETDRS testing, visual field tests, color vision, dynamic pupillometry, and nystagmus testing results will be summarized with appropriate descriptive statistics by gene mutation subgroup, and disease cohort.
- The subject questionnaire will be summarized with appropriate descriptive statistics by gene mutation subgroup, and disease cohort.

Study Duration:

This study started in the fall of 2009 and is expected to be completed in the spring of 2013.

TABLE OF CONTENTS

PROTOCOL APPROVAL 2
PROTOCOL SUMMARY 3
ABBREVIATIONS AND DEFINITIONS 9
1 INTRODUCTION AND BACKGROUND 10
1.1 Pharmacological Class 10
1.2 Retinoids and Vision 10
1.3 Potential Indications and Clinical Development Rationale for QLT091001 11
1.3.1 Disease Characteristics of LCA and RP 12
1.3.2 Proposed Mechanism of Action for QLT091001 12
1.3.3 Other Treatment Options for LCA and RP 14
1.4 Summary of Preclinical Data on QLT091001 15
1.4.1 Vision Rescue 15
1.4.2 Safety Pharmacology 17
1.4.3 Toxicology 17
1.5 Study RET HV 01 (Safety, First in Humans) 18
1.6 Potential Risks and Benefits to Human Subjects 19
2 RATIONALE 19
2.1 Rationale for the Study 19
2.2 Rationale for Drug Dose Selection 20
3 STUDY OBJECTIVES 21
4 INVESTIGATIONAL PLAN 21
4.1 Overall Study Design 21
4.2 Discussion of Study Design 23
4.2.1 Rationale for Selection of Vision/Efficacy Tests 23
5 SELECTION AND WITHDRAWAL OF SUBJECTS 24
5.1 Number of Subjects 24
5.2 Inclusion Criteria 24
5.3 Exclusion Criteria 25
5.4 Withdrawal of Subjects 26
6 RANDOMIZATION AND MASKING PROCEDURES 27
7 STUDY TREATMENTS 28
7.1 Investigational Drug and Device Specifications 28
7.2 Drug Dosage and Administration 28
7.3 Drug Dose Modification 29
7.4 Concomitant Treatment 29
8 RISKS/PRECAUTIONS 29
8.1 Signs and Symptoms of Hypervitaminosis A 30
8.2 Anticipated Clinical Effects Based on Nonclinical Studies 30

8.3 Anticipated Clinical Effects Based on Clinical Studies 31
8.4 Reproductive Risks and Precautions 31

9 STUDY PROCEDURES 31
9.1 Schedule of Events 31
9.2 Screening Procedures 34
9.3 Treatment and Follow-up Procedures 34
9.4 Safety Assessments 34
9.4.1 Pregnancy Testing 34
9.4.2 Vital Signs 35
9.4.3 ECG 35
9.4.4 Clinical Laboratory Tests 35
9.4.5 Height and Weight Measurement 36
9.5 Efficacy Tests 36
9.5.1 Best-Corrected Visual Acuity: ETDRS Testing 36
9.5.2 Color Vision 36
9.5.3 Visual Field Tests 37
9.5.4 Full-field ERG 37
9.6 Other Procedures 37
9.6.1 Blood Samples for Analysis of QLT091001 and Metabolites 37
9.6.2 Dynamic Pupillometry and Nystagmus Testing 37
9.6.3 fMRI (Separate Addendum Protocol; Applies Only to Canadian Study Center) 37
9.6.4 Videography 38
9.7 Subject Questionnaire 38
9.8 Instructions for Subjects 38

10 EVALUATION, RECORDING, AND REPORTING OF ADVERSE EVENTS 39
10.1 Definitions 40
10.1.1 Adverse Event (AE) 40
10.1.2 Serious Adverse Events (SAEs) 40
10.2 Adverse Event Descriptions 41
10.2.1 Intensity 41
10.2.2 Relationship to Study Treatment 41
10.3 Reporting and Evaluation of Serious Adverse Events and Other Clinically Significant Adverse Events 41
10.4 Adverse Event Definitions for Discontinuation Criteria 42
10.5 Follow-up for Adverse Events 42
10.6 Pregnancy Follow-up 43
10.7 Reporting of Technical Complaints about the Investigational Drug 43
10.7.1 Definitions 43
10.7.2 Reporting of Technical Complaints 43

11 STATISTICAL CONSIDERATIONS 44
11.1 Sample Size 44
11.2 Data Sets to be Analyzed 44
11.2.1 Efficacy: Intent to Treat 44

11.2.2 Safety 44
11.3 Analysis of Demographic and Baseline Data 44
11.4 Efficacy Variables and Analyses 45
11.4.1 Best-Corrected Visual Acuity Using ETDRS Testing 45
11.4.2 ERG Variables 45
11.4.3 Goldmann Visual Field (GVF) 45
11.4.4 Other Efficacy Variables 45
11.5 Safety Variables and Analyses 46
11.6 Other Analyses 46
11.6.1 Subject Questionnaire 46
11.6.2 QLT091001 Levels 46

12 ESTIMATED DURATION OF THE STUDY 46

13 STUDY ETHICAL CONSIDERATIONS 47
13.1 Ethical Conduct of the Study 47
13.2 Informed Consent and Assent 47
13.3 Institutional Review Board, Ethics Committee, or Research Ethics Board (IRB) 47

14 ADMINISTRATIVE PROCEDURES 48
14.1 Sponsor's Responsibilities 48
14.1.1 Study Supplies 48
14.1.2 Investigator Training 48
14.1.3 Ongoing Communication of Safety Information During the Study 48
14.1.4 Study Monitoring 48
14.1.5 Records Retention 49
14.2 Investigator's Responsibilities 49
14.2.1 Reporting and Recording of Study Data 49
14.2.2 Source Documentation 49
14.2.3 Study Drugs 50
14.2.4 Records Retention 50

15 POLICY FOR PUBLICATION AND PRESENTATION OF DATA 50

16 REFERENCES 51

ABBREVIATIONS AND DEFINITIONS

AE	Adverse event
ALT	alanine aminotransferase
AST	aspartate aminotransferase
APTT	activated partial thromboplastin time
BCVA	best-corrected visual acuity
BSA	Body surface area
BUN	blood urea nitrogen
CI	confidence interval
CRF	Case report form
CVFQ	Children's Visual Function Questionnaire
ECG	Electrocardiogram
ETDRS	Early Treatment Diabetic Retinopathy Study
ERG	Electroretinogram
EU	European Union
FA	fundus albipunctatus
FAF	fundus autofluorescence
fMRI	functional magnetic resonance imaging
HFA	Humphrey's Visual Field Analyzer
GCP	Good Clinical Practice
HDL	high density lipoprotein
HPLC	high-performance liquid chromatography
HRR	Hardy-Rand-Rittler
IB	Investigator's Brochure
ICH	International Conference on Harmonization
IOP	intraocular pressure
IRB	Institutional Review Board, Ethics Committee, or Research Ethics Board
IRD	Inherited retinal disease
ITT	Intent-to-treat
IU	International units
IUD	intrauterine device
KDa	kilodalton
LCA	Leber Congenital Amaurosis
LDL	low density lipoprotein
LLQ	Low Luminance Questionnaire
LRAT	lecithin:retinol acyltran*s*ferase
LRAT	human gene encoding the protein lecithin:retinol acyltransferase
Lrat	murine *LRAT* gene
MedDRA	Medical Dictionary for Regulatory Activities
mv	millivolt
NOAEL	No observable adverse effect level
OCT	Optical coherence tomography
ONL	Outer nuclear layer
PAD	Pharmacologically active dose
PK	Pharmacokinetics
PT/INR	prothrombin time–international normalized ratio
RP	retinitis pigmentosa
RPE	retinal pigment epithelium
RPE65	retinal pigment epithelium-specific protein 65 KDa
RPE65	human or canine RPE65 gene
Rpe65	murine *RPE65* gene
SAE	Serious adverse event
T3	triiodothyronine
T4	thyroxine
TdP	torsade de pointes
TG	triglycerides
TSH	thyroid stimulating hormone
US	United States
V_{max}	Maximum initial velocity of enzyme-catalyzed reaction at saturating substrate levels
WPW	Wolff-Parkinson-White

1 INTRODUCTION AND BACKGROUND

This will be a phase 1b study to investigate the safety, tolerability, and proof-of-concept for oral QLT091001 as a new treatment for subjects with Leber Congenital Amaurosis (LCA) or retinitis pigmentosa (RP) due to inherited mutations in *RPE65* (encoding the protein retinal pigment epithelial protein 65) or *LRAT* (encoding the enzyme lecithin:retinol acyltransferase). The study will be conducted in compliance with the protocol, International Conference on Harmonization (ICH) good clinical practice (GCP) guidelines, and Part C, Division 5 of the Canadian Food and Drug Regulations.

Subjects with either LCA or RP will be enrolled in 1 study center in Canada, a maximum of 5 subjects with RP will be enrolled at approximately 2-3 study centers in the United States, and adult subjects with RP will be enrolled at approximately 3 study centers in the European Union.

In Canada, this protocol is associated with addendum protocol RET IRD 01 (fMRI), which describes functional magnetic resonance imaging (fMRI) procedures to be done in conjunction with this main protocol, if feasible and if separate informed consent is obtained. Please refer to addendum protocol RET IRD 01 (fMRI) for more information.

Study RET IRD 01 will be associated with a subsequent retreatment protocol, which will enroll subjects from RET IRD 01 who meet entry criteria for retreatment.

1.1 Pharmacological Class

QLT091001 is a retinoid, which as a class has similarity to the structural and/or biological properties of Vitamin A. Vitamin A has several important physiological functions including vision, growth and differentiation of epithelial tissue, bone growth, reproduction and embryonic development [1].

There are currently several synthetic oral retinoid drugs approved in Canada, the United States (US), and the EU for dermatologic or oncologic indications: Accutane/Roaccutane® or Isotretinoin Capsules (isotretinoin; 13-*cis*-retinoic acid) for the treatment of severe recalcitrant nodular acne [2,3], Soriatane® or Neotigason® (acitretin; an all-trans isomer derivative of retinoic acid) for the treatment of severe psoriasis [4,5], Vesanoid® or Tretinoin Capsules (tretinoin; all-trans-retinoic acid) for the treatment of acute promyelocytic leukemia [6,7], and Targretin® (bexarotene; a structurally unique retinoid) for the treatment of cutaneous manifestations of T-cell lymphoma [8].

1.2 Retinoids and Vision

The important Vitamin A derivative involved in vision is 11-*cis*-retinal, which is produced in and by the retinal pigment epithelium (RPE) from the isomerization and oxidation of all-*trans*-retinol (Vitamin A derived from the diet) [9]. In the rod system, 11-*cis*-retinal functions as a chromophore and binds to the protein opsin to form rhodopsin in the rod outer segment. 11-*cis*-retinal is very unstable unless bound to opsin. Vision is initiated when a light photon is captured by 11-*cis*-retinal, resulting in isomerization to all-*trans*-retinal and

dissociation from opsin. Vision is sustained by the cycling of all-*trans*-retinal back into 11-*cis*-retinal, which occurs by a complex series of biochemical reactions involving multiple enzymes and proteins in the retinoid cycle (also known as the vitamin A cycle). Briefly, all-*trans*-retinal is reduced to all-*trans*-retinol in the rod outer segment and then transferred to the RPE layer of the retina. In the RPE, all-*trans*-retinol is esterified to all-*trans*-retinyl palmitate, then simultaneously hydrolyzed and isomerized to 11-*cis*-retinol. Finally, 11-*cis*-retinol is isomerized back into to 11-*cis*-retinal. Figure 1 depicts this process and the enzymes and proteins involved.

FIGURE 1. Rod-Specific Retinoid Cycle

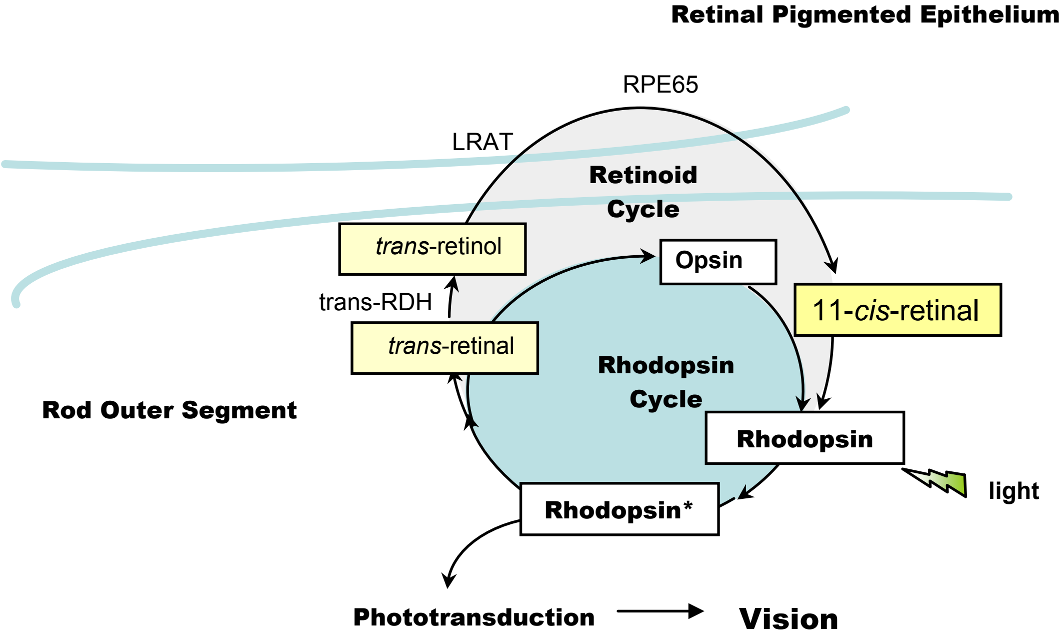

Disruption in the expression of any one of the essential enzymes driving these fundamental processes and/or defects in the structural elements RPE or Bruch's membrane will lead to visual dysfunction. Furthermore, mutations in the *RPE65* and lecithin retinol acyltransferase (*LRAT*) genes are associated with severe, early onset retinal dystrophy. *RPE65* is the gene that encodes retinal pigment epithelium-specific 65-kD protein [10,11], and *LRAT* is the human gene encoding the protein that catalyzes esterification of all-trans-retinol into all-trans-retinyl ester in the phototransduction process, which is a necessary step in the retinoid cycle [12].

1.3 Potential Indications and Clinical Development Rationale for QLT091001

The investigational drug QLT091001 is under development for inherited retinal diseases (IRD) caused by gene mutations that disrupt or interfere with the production, conversion and/or regeneration of 11-*cis*-retinal, resulting in severe visual impairment and blindness, such as the IRDs LCA and RP. These IRDs of interest are rare retinal diseases including subtypes of LCA: RP, and fundus albipunctatus (FA). Several gene defects have been linked

to each of these IRDs; however, mutations in retinoid cycle genes that result in biochemical defects for which therapy with QLT091001 may provide benefit include both retinal pigment epithelium protein 65 (*RPE65*) and lecithin:retinol acyltransferase (*LRAT*).

1.3.1 Disease Characteristics of LCA and RP

As identified in Figure 1, LRAT and RPE65 enzymes are sequentially involved in the regeneration of 11-*cis*-retinal from all-*trans*-retinol in the RPE. LRAT is responsible for the formation of retinyl esters that are substrates for the isomerization and hydrolysis reactions needed to create 11-*cis*-retinal [13]. RPE65 is an essential component required for isomerization of retinyl esters and has been previously suggested to play a role in presenting retinyl esters to the isomerohydrolase [14]. Recent reports suggest that RPE65 itself is the isomerohydrolase, and requires co-expression of LRAT for in-vitro activity [15,16,17]. Mutations in the *RPE65* and *LRAT* genes are associated with vision loss in LCA or RP. Other mutations in other retinal genes have also been identified in separate genetic subtypes of LCA and RP; however, these are not related to defects in the production of 11-*cis*-retinal.

The clinical characteristics and progression of disease in LCA or RP are distinct but also overlap, as do some of their genetic causes. Indeed, 7 of the 15 known LCA disease genes, including *CRX*, *CRB1*, *RPE65*, *RDH12*, *LRAT*, *MERTK*, and *TULP1*, have also been linked to the clinical appearance of RP in other families [18,19,20]. A common characteristic of the IRDs caused by *LRAT* or *RPE65* mutations is early onset night blindness. LCA is the most severe form of all IRDs and has features of congenital and severe visual loss, wandering nystagmus, abnormal pupillary responses (amaurotic pupils), a severely reduced or abolished electroretinogram (ERG), significant hyperopia (farsightedness), and retinal appearances ranging from near normal to a large variety of pigmentary changes [18,21]. RP is a retinal dystrophy with several phenotypical overlaps with LCA, but is initially much milder and with a much older age of onset but that also results in progressive visual loss [22]. It is thought that LCA accounts for 5% of all retinal dystrophies but this prevalence may be underestimated due to under-reporting and diagnostic difficulties.

LCA exhibits clinical and genetic heterogeneity in terms of the natural history of vision loss, behavior in low light conditions, and genetic defects responsible for the phenotype. Due to the significant clinical overlap and some overlap in the responsible gene defects of LCA and RP, some physicians describe these two conditions as essentially the same condition at opposite ends of the severity spectrum. As a general, but not universal, rule the diagnosis of RP or LCA is usually given based on the age of onset, with LCA commencing at a much earlier age than RP [22].

1.3.2 Proposed Mechanism of Action for QLT091001

Despite disease heterogeneity and terminology, there is overlap in the genetic mechanisms underlying some forms of LCA and RP, such as those caused by *RPE65* and *LRAT* mutations where 11-*cis*-retinal production is either severely or completely compromised. Synthetic retinoid 9-*cis*-retinyl ester 'replacement' therapy is proposed for this shared metabolic

disturbance. This study will evaluate QLT091001 as a potential new treatment option for subjects with LCA or RP due to RPE65 or LRAT deficiency.

The therapeutic strategy with 9-*cis*-retinal is to rescue vision by acting as a replacement for 11-*cis*-retinal and restoring the key biochemical component of the visual (retinoid) cycle. The 9-*cis*-isomer of retinal forms the visual pigment isorhodopsin (instead of the usual rhodopsin, Figure 2), and when this visual pigment is bleached, it undergoes conformational changes through the same photoproducts as 11-*cis*-retinal regenerated rhodopsin, and is thermodynamically more stable than 11-*cis*-retinal [10]. In addition, isorhodopsin has an absorption maximum hypochromicly shifted by 8 nm (lambda = 494 nm), compared to rhodopsin (lambda = 502 nm). Furthermore, 9-*cis*-retinal injected into vitamin A-deprived rats has been shown to result in the formation of isorhodopsin [23]. It is hypothesized that in humans with a deficiency in 11-*cis*-retinal, isorhodopsin may act as a suitable substitute for rhodopsin in the visual cycle [23,24]. This strategy is supported by studies in *Rpe65*-deficient mice that detected a restoration of ERG measures after supplementation with QLT091001 [10,23, unpublished].

FIGURE 2. Schematic Drawing of the Structure of the Chromophore in Rhodopsin, Bathorhodopsin, and Isorhodopsin

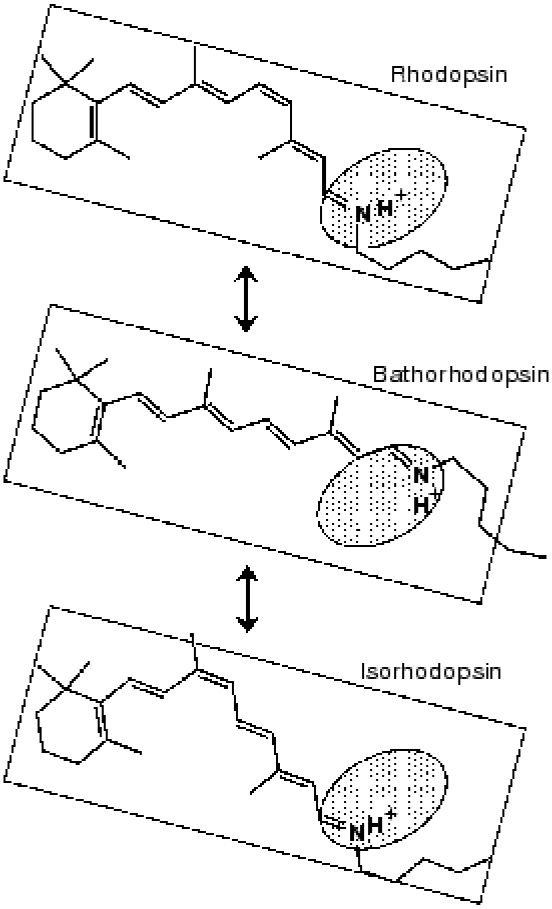

(Modified from Kandori and Maeda [25])

1.3.3 Other Treatment Options for LCA and RP

There are currently no approved treatments for LCA or RP in North America, and only one treatment is approved in the the European Economic Area (Argus™ II Retinal Prosthesis System, an implantable device intended to treat profoundly blind people with degenerative diseases such as RP [26]).. A limited number of therapies are in development, all of which are invasive and require surgical implantation of prosthetic retinal chips or subretinal injection. Gene therapy is one possible treatment being investigated for LCA, using a subretinal injection to deliver an adeno-associated virus as the vector, which contains the wildtype cDNA of *RPE65* with a retinal promoter. Three recent human studies have reported on the responses of visual function to gene replacement for LCA subjects with *RPE65* gene defects, showing variable success. In the first study, 1 of 3 LCA subjects with *RPE65* mutations (age 17-23 years) had significant improvements in retinal sensitivity on dark-adapted perimetry, and improved visual mobility in a simulated street scene [11]. However, there were no changes in visual acuity, peripheral fields (as measured by Goldmann kinetic perimetry), or ERG in any of the 3 subjects. In the second study, all 3 LCA subjects (age 19-26 years) with *RPE65* mutations showed significant improvements based on pupillary light reflexes, best-corrected visual acuities using Early Treatment Diabetic Retinopathy Study (ETDRS), and a reduction in nystagmus frequency. One of the 3 subjects also exhibited a remarkable improvement in visual mobility during an obstacle course [27]. In the third study, 3 LCA subjects (age 21-24 years) showed a statistically significant increase in visual sensitivity, localized to retinal areas that had received the adeno-viral vector containing *RPE65* cDNA, at 30 days after treatment [28,29]. Upon testing by rod- and cone-specific automated perimetry, both cone- and rod-photoreceptor-based vision could be detected in the treated areas. For cones, the increase in retinal sensitivity was 50-fold while for rods there was a 63,000-fold increase in sensitivity.

Although no adverse effects were found in these short-term studies (including immunological events), subretinal injections carry a potentially significant risk, including retinal tears or retinal detachment, hemorrhage, infection, and inflammation, which could all lead to permanent loss of any remaining functional vision in these subjects. The importance of the previous human experimental gene replacement studies is that they show proof of the principle that photoreceptor cells, though not participating in active vision in the *RPE65* defective LCA retinas, are still alive and metabolically active and are able to respond to external rescue. Also, although dormant, the retinoid cycle is able to respond when the missing intermediate (in this case the RPE65 protein) is added. These experiments bode extremely well for the planned experiments in this proposal, namely to stimulate the retinoid cycle by an oral vitamin A intermediate [30]. The results of these exciting human gene therapy studies suggest that some degree of visual function may be restored in subjects with LCA. It remains to be seen whether better treatment outcomes could be achieved with gene therapy if administered at an earlier age and earlier stage of retinal disease. Given the promising results, yet potentially significant risks, of gene replacement therapy, it is worthwhile and logical to evaluate potential alternative treatments for LCA and RP such as oral QLT091001 to achieve the same bypass of the block in the retinoid cycle. If found to be efficacious, this treatment may provide a non-invasive therapy with a broad target subject population (i.e., both *RPE65* and *LRAT*-deficient subtypes of LCA and RP).

1.4 Summary of Preclinical Data on QLT091001

In this clinical trial, the investigational product is an oral formulation of QLT091001 prepared in soybean oil for metered dosing. QLT091001 is a prodrug that is converted by hydrolysis in vivo to 9-*cis*-retinol.

A comprehensive program of nonclinical studies has been completed to support this clinical study, including pharmacology, pharmacokinetic, single- and repeat-dose toxicology, and genotoxicity studies. The most relevant findings from these studies are summarized in the following sections. For more complete information, please refer to the Investigator Brochure (IB) for QLT091001.

1.4.1 Vision Rescue

< preclinical experience with QLT091001 redacted >

< preclinical data redacted >

< *preclinical data redacted* >

1.4.2 Safety Pharmacology

< *preclinical data redacted* >

1.4.3 Toxicology

< *preclinical data redacted* >

< preclinical data redacted >

1.5 Study RET HV 01 (Safety, First in Humans)

Study RET HV 01 was conducted to investigate the safety and tolerability of daily oral doses of QLT091001 for 7 days in 6 dose level cohorts of healthy adult volunteers. Three subjects were included in each dose cohort with the exception of the highest dose cohort which included 5 subjects. Doses escalating from 1.25 mg/m^2 to 40 mg/m^2 were tested in series approximately 2-3 weeks apart through a doubling process (1.25 to 2.5 to 5.0 to 10 mg/m^2 etc.). For each dose cohort, extensive laboratory tests and vital signs were evaluated at Screening, the evening before dosing, the next morning shortly before the initial dose, and at regular intervals during and after the 7-day dosing period. AEs were evaluated at every visit beginning on Day 1. Holter monitoring was done at Screening, 24 hours following the first dose, and the day after the last dose; ECGs were done at Screening, the day before the initial dose, before and after dose 3, and 3 days after the last dose. When all test results up to 3 days after the seventh dose were available for a specific cohort, a safety monitoring committee reviewed the information before dose escalation was allowed for the next cohort.

In the cohorts dosed with 10 to 40 mg/m^2, a reduction of plasma high density lipoprotein (HDL) concentrations was observed in most subjects. This HDL reduction did not appear to be dose-dependent and was rarely out of the normal-low range. Some of these subjects also had milder reductions of plasma low density lipoprotein (LDL) concentrations. Isolated reductions in one or more hematologic parameters (hemoglobin, red blood cells, hematocrit) were also noted in 2 subjects (1 subject who took the 5 mg/m^2 dose and 1 who took the 40 mg/m^2 dose) but these events were mild and not considered to be clinically significant. Drug-related adverse symptoms only occurred in the 5 subjects receiving 40 mg/m^2 who all

reported mild headache(s); some subjects also reported mild facial flushing (2/5) and mild loss of appetite (2/5) and 1 subject reported moderate nausea. These AEs were noted during the first 4 days of dosing and resolved thereafter.

Overall, 7 daily oral doses of QLT091001 up to 40 mg/m^2 were well tolerated. A more complete description of this study is provided in the Investigator Brochure (IB).

1.6 Potential Risks and Benefits to Human Subjects

The main risks of QLT091001 and 9-*cis*-retinol in humans are unknown but, at the dose and treatment duration planned for this study, may include adverse effects on the liver based on rat and primate toxicology studies and human experience with other oral retinoid drugs [33,40,41,42]. These effects will be monitored by serum chemistry analysis and are typically reversible. These and other risks in humans are described further in Section 8, Risks/Precautions. For more detailed information, please refer to the IB for QLT091001. The risks of daily oral dosing of QLT091001 for 7 days in humans were investigated in a phase 1a healthy volunteer study, RET HV 01 (Section 1.5).

The potential benefits of QLT091001 are the following:

- The ability to produce isorhodopsin within the photoreceptors should result in improved vision in subjects who have retained some photoreceptors and retinal architecture.
- Preservation of some visual function by this method may also prevent the progressive degeneration of the retina that is observed in these subjects.

2 RATIONALE

2.1 Rationale for the Study

LCA and RP are relatively rare inherited diseases associated with blindness at birth and slowly progressive retinal degeneration, with further decline in visual function. Two to three per 100,000 newborns have LCA [43], and we estimate that 200,000 LCA cases exist in the world. The world-wide prevalence of RP is estimated to be approximately 1 in 4000 [44]. Between 6%-16% of LCA subjects and approximately 2% of RP subjects have mutations in *RPE65* and approximately 1% of LCA and RP subjects harbor mutations in *LRAT*. Mutations in these two genes result in RPE65 and LRAT enzyme deficiency, which results in a deficiency or absence of 11-*cis*-retinal and subsequently in rhodopsin (11-*cis*-retinal + opsin).

In animal gene knockout models of these enzyme deficiencies, oral administration of QLT091001 has resulted in improved visual function [10,23]. The presumed mechanism for this improvement is the provision of a depot of 9-*cis*-retinol within the RPE that can be metabolized to 9-*cis*-retinal, which can subsequently function as the visual chromophore when combined with opsin to form isorhodopsin in the photoreceptor outer segments. This current study is being done to see if the successful results of the preclinical animal models can be reproduced in human subjects with the same condition.

In LCA and RP subjects, while the exact nature and time course of retinal degeneration depends on the genetic defect and type of mutation, the viability of treatable retinal tissue (and the number and viability of photoreceptors) is thought to decrease with time (and age) and the earliest treatment intervention should lead to the best outcomes, when the outer nuclear layer (ONL) which houses both rod and cone photoreceptor cell bodies is thickest. Thus, for this treatment to be of clinical benefit in subjects with LCA, it should be started as soon as possible after diagnosis, likely the first year of age, and continued indefinitely. It is unknown what the upper age for successful treatment would be, and it may vary from case to case. Because this clinical study is primarily intended to assess if the treatment may be of any clinical benefit, the study subjects to be included should have as much retinal integrity and as many viable photoreceptors as possible and therefore, be as young as practically possible but be old enough to read and provide feedback from visual tests using letter charts. Consequently, the minimum age of 5 years was selected for subjects with LCA because, at this age, children should be old enough to read letters, which is necessary to complete the efficacy assessments, or to follow directions for tracing the letters. Recent experience in gene therapy trials have demonstrated that some degree of treatment success is also possible in adult subjects up to the fourth decade [45]; therefore, adult subjects will also be eligible for this study as long as they exhibit evidence of a viable photoreceptor layer, indicating a reasonable chance of benefit from treatment with QLT091001. (Optical coherence tomography [OCT] and fundus autofluorescence [FAF] will be performed on all subjects during the Screening period to confirm there is a viable photoreceptor layer in the retina.)

2.2 Rationale for Drug Dose Selection

This study is to establish proof of principle. The dose of 40 mg/m^2 to be used in this study is the maximum dose assessed in healthy volunteers (study RET HV 01). The range of doses studied in RET HV 01 (1.25 to 40 mg/m^2) includes the expected pharmacologically active dose (PAD) range for LCA and RP of between 3 to 37.5 mg/m^2/day. This range is estimated based on a 14-day study of QLT091001 in a genetic mouse model of LCA (*Rpe65* $^{-/-}$), in which 3 mg/m^2/day produced a small, detectable effect on ERG response (1 mg/kg/day). However, a stronger response was seen at 12 and 37.5 mg/m^2/day (4 and 12.5 mg/kg/day). 9-*cis*-retinal or 9-*cis*-retinyl esters were detected in the retina only at the two higher doses. Please refer to Section 1.4.1 above for more information about this in vivo pharmacology study.

The 40 mg/m^2 dose can be viewed as a loading dose to maximize the chances of observing an effect and meeting the goal of establishing proof of principle. Based on the first study in humans, this dose is expected to be safe and well tolerated.

The 40 mg/m^2 dose is considered reasonable because manifestation of retinoid toxicity is more dependent on dosing duration than acute dose magnitude. Furthermore, most signs and symptoms are reversible on dosing cessation. The Sponsor will carefully monitor patients through serum chemistry and other clinical evaluations to assess any emerging signs and symptoms. Should any safety concerns or tolerance issues arise, the dose may be reduced at the Investigator's discretion. Refer to Section 7.3 for details on drug dose modification.

Notes:

- Based on results of metabolic studies in *Lrat*-deficient mice, it is possible that subjects with the LRAT mutation may be less sensitive to treatment because they may not be able to store 9-*cis*-retinyl esters, the storage form of 9-*cis*-retinol, in their eyes and most tissues [46,47]. Consequently, these subjects may need a higher dose to respond. These subjects may also tolerate the higher dose better than subjects with the other mutations.

- This will be the first time QLT091001 is administered to children, thus there are no data to indicate if there is a difference in the pharmacokinetics (PK) of study drug in pediatric compared with adult subjects. However, the drug Accutane,® which is a retinoid like QLT091001, exhibits no differences in PKs between adults and children. Thus, no differences in PKs are anticipated when administering study drug to children at the highest dose used in the adult study. See Accutane label for reference [2,3].

3 STUDY OBJECTIVES

- To evaluate whether 7-day treatment with oral QLT091001 can improve visual function in subjects with LCA or RP caused by *RPE65* or *LRAT* gene mutations.
- To evaluate duration of visual function improvement (if observed) in subjects with LCA or RP caused by *RPE65* or *LRAT* gene mutations after 7-day treatment with oral QLT091001.
- To evaluate the safety of oral QLT091001 administered once daily for 7 days in subjects with LCA or RP caused by *RPE65* or *LRAT* gene mutations.

4 INVESTIGATIONAL PLAN

4.1 Overall Study Design

This will be an open-label, proof-of-concept, phase 1b study. The study will include up to 28 subjects with LCA or RP due to inherited deficiency of *RPE65* or *LRAT* (up to 14 subjects for each disease cohort). Subjects in each disease cohort (including 2 gene mutation subgroups) will receive a once-daily oral loading dose of 40 mg/m^2 QLT091001 for 7 consecutive days, which was the maximum dose assessed in study RET HV 01 (see Figure 4). Subjects with either LCA or RP will be enrolled at 1 study center in Canada. A maximum of 5 subjects with RP will be enrolled at 2-3 study centers in the US. Adult subjects with RP will be enrolled at approximately 3 study centers in the EU.

FIGURE 4. Study Design Schematic

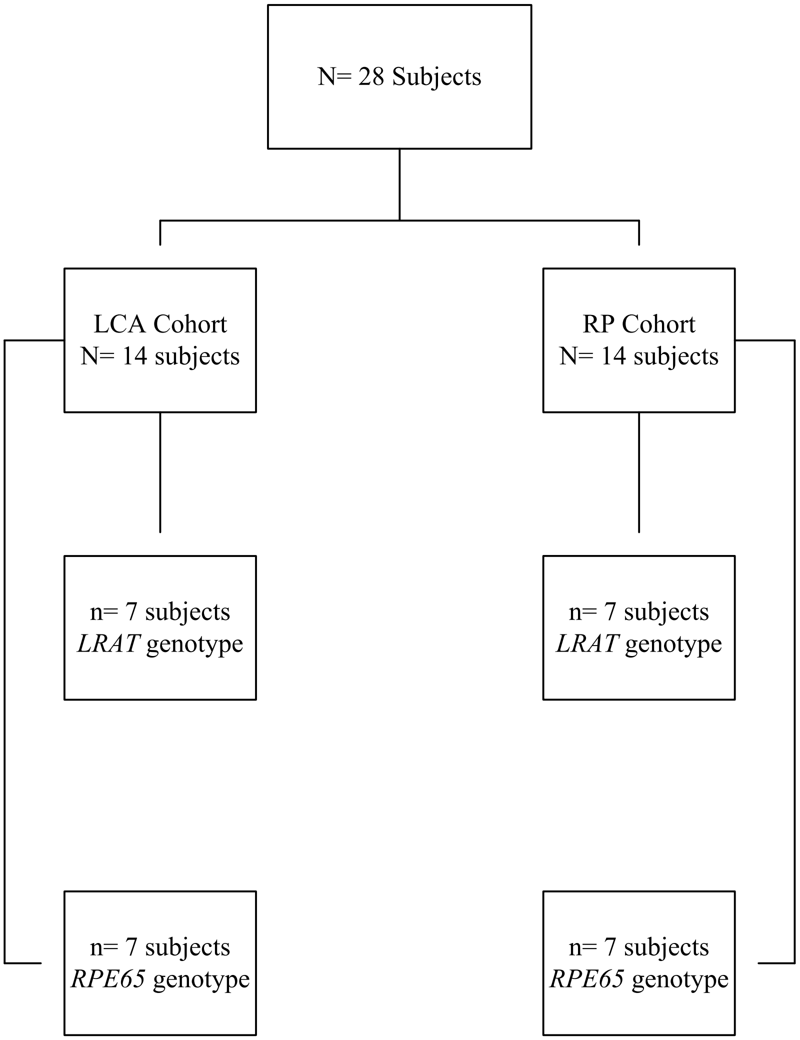

Subjects will be treated on an outpatient basis but will receive study treatment in the research clinic under medical supervision for each day of treatment. During the study treatment period and for 7 days post-treatment, subjects will be required to limit vigorous physical activity and also must follow dietary guidelines intended to avoid excessive vitamin A intake.

To determine proof-of-concept and safety, subjects will have the following ocular examinations (on both eyes) performed before and after treatment:

1. Best-corrected visual acuity (BCVA) using ETDRS (monocular and binocular tests)
2. Color vision

3. Visual field
4. Full-field ERG
5. OCT and FAF
6. Dynamic pupillometry and nystagmus testing (if instrument is available)

Safety evaluations will include monitoring of vital signs, ECG, clinical laboratory tests (12-hour fasting serum chemistry and hematology, serum retinol, coagulation testing, thyroid function testing, and urinalysis), biomicroscopy, IOP, dilated fundus examination, AEs, and concomitant medications.

Additionally, a subject questionnaire will be given to the subject and/or parent/guardian (if applicable) for completion at Screening and at follow-up visits starting at Day 14/15. Follow-up visits will continue through 12 months post-treatment, although subjects will complete the study at the Day 30 visit, or at any visit after that, if they meet entry criteria and are enrolled into a subsequent study for retreatment.

4.2 Discussion of Study Design

The efficacy variables being used for this study are intended to generate results that are relatively stable over the very short time period during which each subject is treated during the study (7 days) and should therefore only change if there is a treatment effect. Subjects will be followed up for at least 12 months after treatment to assess the duration of treatment effect, as well as any potential delayed treatment effect. In preliminary data from this study, beneficial treatment effects have been observed in some subjects in at least 1 vision function measurement, but the duration of treatment effect is unclear, as is the timing of the onset of treatment effect [48]. Beneficial treatment effects need to be clear and clinically relevant if the treatment is to be developed further. The study does not include a control group, so each subject's baseline visual function will serve as the control for that subject.

Each subject will undergo daily treatments for 7 days, as it is believed that it will take at least 7 days for adequate amounts of QLT091001 and its precursor 9-*cis*-retinol to accumulate in the retina. However, a visual acuity test using best-corrected ETDRS is planned 24 hours after the first dose to evaluate whether a clinical effect of treatment occurs more rapidly.

4.2.1 Rationale for Selection of Vision/Efficacy Tests

No single test has been validated to definitively evaluate the response of treatment for LCA or RP. One potential benefit of the vision tests selected for this study will be identification of a primary endpoint for subsequent trials, if a beneficial treatment effect is observed.

While ERG testing is the gold standard for evaluating therapeutic levels of several experimental therapies and is used routinely to diagnose and monitor progression of most inherited retinal diseases including LCA, ERG testing has not yet been correlated with endpoints accepted by regulatory agencies, such as ETDRS vision and Humphrey Visual Field Analyzer (HFA). A limitation of full-field ERG is that the recording is a massed potential from the whole retina. Unless 20% or more of the retina is affected with a diseased

state, ERG recordings are usually normal (e.g., a legally blind person with macular degeneration, enlarged blind spot or other central scotomas has normal global ERGs). Most LCA and RP subjects have virtually no measurable ERG recordings, yet many of these subjects can still see, some quite well. Recent gene therapy trials for LCA have not reported changes in full-field ERG results because the methods in these trials treated less than 10% of the retina, so the ERG results would not be expected to change.

A method for quantitative analysis of Goldmann visual field (GVF) is being developed and has shown promise as an endpoint in subjects with LCA and RP in the current study, as described in the IB. (GVF has been used instead of HFA because HFA is usually not appropriate for young subjects and subjects with very low vision.) This GVF analysis is incorporated into the study for assessment as a potential primary endpoint for future trials (see Section 11.4).

5 SELECTION AND WITHDRAWAL OF SUBJECTS

5.1 Number of Subjects

Up to 28 subjects in total will be enrolled in this study. This includes up to 7 subjects with LCA for each genotype subgroup (*RPE65* or *LRAT* mutations) and up to 7 subjects with RP for each genotype subgroup (*RPE65 or LRAT* mutations). A maximum of 5 subjects with RP will be enrolled in the US. Subjects from outside of North America and Europe are eligible and will be required to travel to a study center to participate in the study.

5.2 Inclusion Criteria

To be eligible for the study, subjects must fulfill all of the following criteria:

1. Subjects will have either LCA or RP, with a mutation in either *RPE65* or *LRAT*, as follows:
    a. Subjects with LCA will be 5-65 years of age diagnosed with LCA by an ocular geneticist or pediatric ophthalmologist.
    b. Subjects with RP will be diagnosed with RP by an ocular geneticist or ophthalmologist and be:

       For Canada, Germany, The Netherlands, and UK: 18-65 years of age (inclusive).

       For US only: 8-65 years of age (inclusive).
2. Subjects who have a best-corrected standard ETDRS visual acuity of **3 letters** or better **(20/800 Snellen equivalent)**, however; subjects with a lower ETDRS score will be eligible if spectral domain OCT and FAF reveals evidence of a viable photoreceptor layer.
3. Subjects who are girls or women of child-bearing potential must not be pregnant or lactating, must have negative serum pregnancy tests (≥25 mIU/mL sensitivity) at Screening (i.e., ≥19 days before Day -1, and on Day -1) and must have been practicing 2 adequate methods of birth control or complete abstinence for at least 2 months.

Adequate methods of birth control include (1) use of oral contraceptives (excluding low-dose oral formulation), implantable or injectable contraceptives, or an intrauterine device (IUD), with an additional barrier method (diaphragm with spermicidal gel OR condoms with spermicide); (2) a double-barrier method (diaphragm with spermicidal gel AND condoms with spermicide); (3) partner vasectomy; and (4) total abstinence.

4. Subjects who are boys or men must (1) agree to completely abstain from sexual intercourse, (2) have had a vasectomy, or (3) use a barrier method (condoms) with spermicide during sexual intercourse, during the treatment phase of the study and for 2 months after finishing the study drug.
5. Subjects who provide informed consent and, if applicable, assent for the study (applies to subjects 7 years and older). The parent or guardian must sign an approved informed consent form for the study for subjects younger than the age of majority.

   Note that use of the term "subject" throughout this protocol may also include the parent/guardian of subjects younger than the age of majority, as appropriate.
6. Subjects who are willing to comply with the protocol.

5.3 Exclusion Criteria

Subjects meeting any of the following criteria will be excluded from the study:

1. Subjects who are actively participating in an experimental therapy study or who have received experimental therapy within 60 days of Day 0.
2. Subjects with any clinically important abnormal physical finding at Screening.
3. Subjects who have taken any prescription or investigational oral retinoid medication (e.g., Accutane/Roaccutane® or Soriatane/Neotigason®) within 6 months of Day 0 and subjects who did not tolerate their previous oral retinoid medication will be excluded regardless of the time of last exposure.
4. Subjects with a history of diabetes or chronic hyperlipidemia, hepatitis, pancreatitis, or cirrhosis.
5. Subjects with liver failure, uncontrolled thyroid disease, hypersensitivity to retinoids, or hypervitaminosis A.
6. Subjects with any of the following findings at Screening:
   - Untreated blood pressure 150/95 mm Hg or higher upon repeated measurement
   - Resting heart rate <40 bpm or >100 bpm upon repeated measurement
   - ALT or AST >3 times the upper limit of the clinical laboratory value normal range upon repeated measurement
   - Total cholesterol, triglycerides, HDL, or LDL >2 times the upper limit of the clinical laboratory value normal range upon repeated measurement
   - Thyroid function tests outside the clinical laboratory value normal range upon repeated measurement

   - Serum retinol clinical laboratory value above 90 µg/dL upon repeated measurement

7. Subjects with a documented and known allergy to soy.
8. Subjects who, in the Investigator's opinion, have any severe acute or chronic medical condition, psychiatric condition, or laboratory abnormality that may increase the risk associated with study participation or administration of study treatment, or interfere with the interpretation of study results.
9. Subjects with a marked baseline prolongation of QT/QTc intervals (e.g., repeated demonstration of a QTc interval >450 milliseconds [ms]).
10. Subjects with a history of additional risk factors for torsade de pointes (TdP) (e.g., heart failure, hypokalemia, history or family history of Long QT Syndrome), and Wolff-Parkinson-White (WPW) syndrome.
11. Subjects who have taken any supplements containing ≥10,000 IU vitamin A within 60 days of Screening.

5.4 Withdrawal of Subjects

Every effort will be made to have all subjects attend all study visits and receive all doses of study drug. Subjects can voluntarily withdraw from study treatment at any time during the study. Every effort will be made to complete study follow-up procedures if subjects terminate the study treatment early. In addition, Investigators may withdraw a subject from study treatment because:

- a new health condition appears that is suspected to require care or medications prohibited by the protocol
- the subject has unacceptable adverse events (AEs)
- the subject has clinical laboratory results as follows:
  - any hematology parameter (as listed in Table 4, Section 9.4.4) that changes by more than 10% above the upper limit of the clinical laboratory value normal range
  - ALT or AST >3 times the upper limit of the clinical laboratory value normal range
  - total cholesterol, triglycerides, HDL, or LDL >2 times the upper limit of the clinical laboratory value normal range
  - thyroid function tests outside the clinical laboratory value normal range
  - serum retinol clinical laboratory value above 90 µg/dL
- the subject has a hypersensitivity reaction to study treatment (immediate withdrawal)
- the subject exhibits clinical signs of hypervitaminosis A, particularly if symptoms occur that indicate an increase in intracranial pressure (immediate withdrawal)
- there is a marked prolongation of the QT/QTc interval, such that

  - it is >500 ms or increases >60 ms from baseline when the measurement obtained is averaged from the triplicate ECGs (see Section 9.4.3) (immediate withdrawal), or
  - in the Investigator's judgment an otherwise marked prolongation may indicate that the subject's safety is in jeopardy.
- it is in the subject's best interest according to the Investigator's clinical judgment

Subjects with the conditions or findings listed above will be considered for withdrawal from study treatment. Withdrawal for the reasons noted above will be reviewed in consultation with the medical monitor (refer to Section 10.3).

If a subject prematurely withdraws from study treatment, the reason(s) for withdrawal must be recorded on the relevant page of the subject's case report form (CRF).

See Section 9 for details on study follow-up procedures and Section 10.5 for details on follow-up for AEs. The Investigator should review the follow-up procedures with the subject and his/her parent(s)/guardian(s) if applicable, including the number of visits, the specific procedures to be done, and the total length of the follow-up period. The Investigator must also ensure it is understood that the subject's medical records will continue to be available for the follow-up period as described in the approved informed consent form for the entire study period.

If a subject refuses to undergo the study follow-up procedures, or his/her parent(s)/guardian(s) refuses to allow the subject to undergo the procedures, the reason for refusal should be fully documented. Subjects who do not continue study procedures should undergo all end-of-study assessments.

Subjects who miss study drug doses will continue to be followed up in the study. Additional subjects may be enrolled to replace subjects who withdraw before completing adequate study follow-up or to supplement data if several subjects miss doses of study drug.

Subjects will withdraw from the study follow-up procedures on Day 30 or at a subsequent visit, and be considered to have completed the study, if they meet entry criteria and are enrolled in a subsequent retreatment study (see Section 1).

The Sponsor may stop the study at any time.

6 RANDOMIZATION AND MASKING PROCEDURES

The study is not randomized or masked.

7 STUDY TREATMENTS

7.1 Investigational Drug and Device Specifications

< QLT091001 description redacted >

7.2 Drug Dosage and Administration

The administration of study treatment was staggered by at least 48 hours between the first and second subjects in the study. (This was completed already at the Canadian study center.)

The dose of 40 mg/m^2 QLT091001 will be administered in this study. This was the highest dose evaluated in a study of healthy adult volunteers (Study RET HV 01).

Dosing will be based on each subject's body surface area (BSA). Each subject's BSA will be calculated using the following formula:

BSA (m^2) = ([Height(cm) x Weight(kg)]/ 3600)^{½} e.g. BSA = SQRT((cm*kg)/3600).

Subjects will have height and weight measured twice (for verification) before dosing, as indicated in Section 9.1 (Table 3). A second qualified person will verify the BSA calculation, the dosing calculation, and preparation of the dose.

Refer to the RET IRD 01 Procedure Manual for complete details regarding dosing calculations.

QLT091001 should be dispensed while wearing gloves using syringes in a room with low lighting.

Subjects will receive 7 consecutive oral loading doses, administered once daily in the morning within 10 minutes after breakfast from Days 0 to Day 6 (inclusive).

Diet and exercise (as well as smoking and drinking) will be restricted. See Section 9.8 for complete details regarding these restrictions.

7.3 Drug Dose Modification

The drug dose may be reduced for a given subject if the proposed dose is poorly tolerated in the clinical Investigator's judgment. Tolerance will be evaluated by monitoring clinical laboratory tests and AEs as defined in the study schedule of events (Section 9.1).

7.4 Concomitant Treatment

Study subjects are not permitted any medications during the treatment period of the study and for 7 days post-treatment, except hormonal contraceptives or if required to manage an adverse event or an ongoing medical issue not precluded by exclusion criteria (Section 5.3). For example, asthma is not precluded by exclusion criteria, so subjects with asthma may enter the study and may need medication during the study; such medication is permitted. As much as possible, such medication should remain at the same dose during the treatment period and for 7 days post-treatment. Any concomitant medications given during the study must be recorded on the CRF and in the source document, including start and stop dates, route, and indication.

Treatment procedures, such as surgical procedures, must be recorded on the CRF and in the source document including start and stop dates. Surgical anesthetics, paramedical or alternative therapies (i.e., acupuncture, massage) should not be recorded on the CRF.

8 RISKS/PRECAUTIONS

The risks of QLT091001 in humans are not known, but may be estimated based on the pharmacological class and observed effects in animal toxicology studies and a recent dosage escalation study in normal human controls.

As a precaution against any unanticipated, severe risk to the administration of QLT091001, the treatment will be administered in a medical facility, with immediate access to equipment and staff for resuscitating and stabilizing individuals in an acute emergency (such as cardiac emergencies, anaphylaxis, cytokine release syndrome, convulsions, hypotension), and in reasonable proximity to Intensive Care Unit facilities.

See Section 1.5 and Section 1.6 for further details about the study conducted in healthy volunteers.

8.1 Signs and Symptoms of Hypervitaminosis A

An overdose of QLT091001 may be expected to present with signs and symptoms similar to chronic vitamin A toxicity (hypervitaminosis A syndrome). Table 2 lists the clinical signs and symptoms that have been reported in subjects with chronic vitamin A toxicity.

TABLE 2. Signs/Symptoms of Hypervitaminosis A Syndrome

Body System	Effects
Mucocutaneous	Dry skin, desquamating rash, pruritus, cheilitis, epistaxis, hair loss, gingivitis, skin fragility and easy bruising, edema, ingrown nails
Ophthalmologic	Blepharitis, papilledema, exophthalmos, diplopia, nystagmus, blurred vision
Neurologic/Psychiatric	Pseudotumor cerebri, headache, depression, psychosis, irritability, fatigue, daytime hypersomnolence, insomnia, lethargy, mood changes
Gastrointestinal	Nausea, vomiting, abdominal pain, anorexia, weight loss, hepatomegaly, splenomegaly, hepatitis, cirrhosis, ascites, varices, hyperlipidemia, elevated liver function tests and alkaline phosphatase
Skeletomuscular	Muscle soreness and stiffness, arthralgia, bone pain, periosteal thickening/calcification, premature closure of epiphyses in children, growth retardation, ossification
Endocrine	Polyuria, polydipsia, abnormal thermoregulation (cold extremities, fever, chills, night sweats)
Other	Elevated prothrombin time (due to vitamin A inhibition of vitamin K absorption), teratogenicity

Source: [39]

The vitamin A doses that are associated with chronic toxicity in a given individual can vary greatly, but generally, doses that exceed normal dietary intake by 15,000 IU/day—or 5 times the recommended daily amounts of 700 µg/day for women and 900 µg/day in men [49]—over several months to years are potentially associated with hypervitaminosis A [39]. Signs and symptoms of hypervitaminosis A typically resolve without long-term sequelae within 2-8 weeks of discontinuing supplementation. However, the reversibility of hepatic or bone effects is less predictable, with some subjects continuing to display abnormal liver function tests or cirrhosis. Teratogenic effects, of course, are also a permanent consequence of vitamin A toxicity.

8.2 Anticipated Clinical Effects Based on Nonclinical Studies

The NOAEL in a monkey toxicology study with 28 days of dosing was 5 mg/kg or 60 mg/m^2 (most relevant species for comparison to humans). Findings at this dose were limited to changes in liver function tests that were not considered clinically significant, did not lead to any changes in treatment, and were reversible after the 28-day treatment course was completed [50].

The target organs for toxicity at the doses <u>above</u> the NOAEL in nonclinical studies were:

- Liver – potential effects will be monitored in this study with serum concentrations of liver enzymes and lipids.

- Bone – potential effects will be monitored in this study by monitoring serum concentrations of alkaline phosphatase and inorganic phosphate. Adverse effects on bone are considered unlikely given the short exposure period in this study.
- Heart – potential cardiac effects will be monitored in this study with ECG and vital signs.

8.3 Anticipated Clinical Effects Based on Clinical Studies

See Section 1.5 for a description of results from the RET HV 01 study. In the current ongoing study (RET IRD 01), preliminary safety data from a total of 14 subjects treated with QLT091001 (12 received 40 mg/m^2 and 2 received 10 mg/m^2) was assessed. In these studies, adverse effects included the following, which may be potential risks with the study drug:

- GI Disorders: nausea
- General Disorders and Administration Site Conditions: asthenia, facial flushing
- Metabolism and Nutrition Disorders: decreased appetite
- Nervous System Disorders: facial burning sensation, headache, photophobia, dizziness
- Investigations: hemoglobin decreased, RBC count decreased, increased triglycerides, decreased HDL, increased or decreased LDL, increased cholesterol, increased AST

8.4 Reproductive Risks and Precautions

Reproductive toxicity studies have not yet been performed with QLT091001. However, QLT091001 is a retinoid and teratogenic effects have been described for this class of compounds. Therefore, subjects will be required to take appropriate precautions. The risk of potential teratogenicity is reduced in this study given the short treatment exposure period. The risk of teratogenicity will be managed similar to the principles in the risk management programs for other oral retinoids (Accutane/Roaccutane® or Soriatane/Neotigason®), as appropriate for this study.

- Girls and women of child-bearing potential must have a negative pregnancy test result prior to being enrolled into the study. Refer to Section 9.4.1 for pregnancy testing requirements.
- The Investigator must provide age-appropriate counseling to all study subjects who may be sexually active on the risk of teratogenicity and how to prevent pregnancy. Subjects will be advised to continue precautions to prevent pregnancy for at least 2 months after completion of the study drug.

9 STUDY PROCEDURES

9.1 Schedule of Events

Table 3 presents the schedule of events for the study.

TABLE 3. Schedule of Events

Procedure[a]	Screening	Study Day											Study Month
	Day –21 to –3	-2/-1	0	1	2	3	4	5	6	7/8[b]	14/15[c]	30 (±4)[c]	2, 4, 6, 8, 10, 12 (±2 wks)
Informed consent	X												
Demography	X												
Inclusion/Exclusion Criteria	X	X											
Medical history	X												
Complete physical exam	X												
OCT Spectralis and FAF[d]	X									X		X	X[e]
Symptom-directed history and physical examination		X	X	X	X	X	X	X	X	X	X	X	X
Blood sample for genetic test[f]	X												
Pregnancy test[g]	X	X									X	X	X[h]
Concomitant medications	X	X	X	X	X	X	X	X	X	X	X	X	X
Chemistry, hematology, thyroid, and serum retinol tests[i]	X	X				X				X	X[j]	X[j]	X[j]
Coagulation testing	X	X				X				X	X[j]		
Urinalysis	X	X				X				X	X[j]		
Vital signs[k]	X	X	X	X	X	X	X	X	X	X	X		
ECG (12 lead)[l]	X	X				X[m]				X			
BCVA	X	X[n]	X[n]	X						X	X	X	X
Color vision test (see Section 9.5.2)	X	X[o]								X	X	X	X
Visual field testing (see Section 9.5.3)	X	X								X	X	X	X
Biomicroscopy	X									X	X	X	X
IOP and dilated fundus exam	X									X	X	X	X
Cycloplegic refraction	X									X[p]	X[p]	X[p]	X[p]
Full-field ERG	X	X								X[p]	X[p]	X	X[p]
Dynamic pupillometry and nystagmus testing[q]		X								X	X	X	X[p]
Subject questionnaire (selected questions from CVFQ or LLQ; see Section 9.7.)	X										X	X	X
PK Sample		X	X[m]	X[i]	X[m]	X[i]			X[m]	X[i]			
Height and weight measurement			X[r]										X[s]
Study treatment (after dose calculation)			X[t]	X	X	X	X	X	X				
Adverse events (AEs)			X	X	X	X	X	X	X	X	X	X	X
Videotaping of treatment-related changes (see Section 9.6.4)[p]	X	X								X	X	X	X
Study completion if enrolled in extension study												X	X

[a] Study procedures will be recorded in the source documents and CRF except physical exam and pregnancy test results, which are only recorded in source documents.

[b] Subjects who discontinue treatment prematurely should undergo all assessments scheduled for the Day 7/8 and the subject questionnaire.
[c] If a subject prematurely withdraws from the study after Day 7/8, the subject should complete the procedures for Day 14/15. If a subject prematurely withdraws from the study after Day 14/15, the subject should complete the procedures for Day 30.
[d] If multiple assessments are done on the same day, OCT Spectralis and FAF must be the last procedures of the day.
[e] OCT Spectralis and FAF will be done at Month 2 and thereafter at the discretion of the Investigator.
[f] Only if genetic testing to confirm the *LRAT* or *RPE65* mutation was not previously done by a fully accredited genotyping laboratory.
[g] For women and girls of child-bearing potential, consent and Screening serum pregnancy test must be performed ≥19 days before Day -1. A serum pregnancy test will also be done on Day -1. A urine pregnancy test will be done on Day 14/15, Day 30, and Month 2. See Section 9.4.1 for further details.
[h] Month 2 only.
[i] Blood samples collected predose on treatment days after a 12-hour overnight fast.
[j] Only if clinically significant laboratory results were observed at the previous visit.
[k] Includes heart rate, resting blood pressure, respiratory rate, and temperature and collected after a 3-minute resting period. Vital signs will be recorded predose and 4 hours postdose on treatment days.
[l] Perform triplicate ECG recordings (3 readings in succession approximately 1 minute apart).
[m] 4 hours postdose.
[n] Two baseline BCVA tests will be done on separate days (Day -2, Day -1 and/or on Day 0 before treatment is given).
[o] Color vision test can be done on Day -2/-1 or on Day 0 before treatment is given.
[p] At the Investigator's discretion.
[q] Dynamic pupillometry and nystagmus testing if appropriate instrument(s) is available.
[r] Pre-dose measurement for BSA calculation for study drug dose.
[s] At the last visit only: height only, measured for subjects ≤19 years of age only.
t Dose must be calculated and study treatment prepared (see Section 7.2), before study treatment is administered.

9.2 Screening Procedures

Study specific screening procedures, as identified in Table 3, will not be performed until after the informed consent form has been signed but within 21 days before Day 0 of the study.

Subjects will undergo a spectral OCT and FAF in low light during the Screening period (as well as the other visits indicated in Table 3) to determine viable photoreceptors in the retina.

Each subject must have a genetic test confirming the mutation (*RPE65* or *LRAT*) from a fully accredited genotyping laboratory. If a subject has genetic test results that confirm the mutation (*RPE65* or *LRAT*), but the results are not from a fully accredited laboratory, the subject must have blood drawn for another genetic test, to be analyzed by a fully accredited genotyping laboratory.

See the RET IRD 01 Procedure Manual for complete instructions for OCT and FAF procedures.

9.3 Treatment and Follow-up Procedures

Treatment and follow-up procedures will be done as identified in Table 3.

On Day 0, each subject will receive the first dose of study drug. Treatment will be administered for 7 consecutive days (Day 0 to Day 6, inclusive).

Subjects will have follow-up visits on Days 7/8, 14/15 and 30; and Months 2, 4, 6, 8, 10, and 12. All visual function and safety assessments will be done on Day 7/8 (24/48 hours after taking the last dose of study drug). Visual function tests and safety assessments will be done on Day 14/15 and each subsequent visit. Women and girls of child-bearing potential will have urine pregnancy tests on Day 14/15, Day 30, and Month 2. AEs and concomitant medications will be monitored at every visit. The subject questionnaire will be completed on Day 14/15 and every subsequent visit. These procedures are described further in Sections 9.4, 9.5, 9.6, and 9.7.

All efforts will be made for subjects to comply with the follow-up visit schedule. However, if the schedule is not feasible for a subject, follow-up visits will be conducted as closely to the schedule as possible.

Subjects will complete the study at the Day 30 visit, or any subsequent visit, if they meet entry criteria and are enrolled into a subsequent retreatment study (see Section 1).

9.4 Safety Assessments

9.4.1 Pregnancy Testing

All girls and women of child-bearing potential will be tested for pregnancy at least 19 days before Day -1. Serum pregnancy tests will be conducted at Screening and on Day -1; a urine pregnancy test will be conducted on Day 14/15, Day 30, and Month 2, as indicated in

Table 3. Pregnancy tests must have a sensitivity of at least 25 mIU/mL. The timing of the Screening pregnancy test must meet the following requirements:

- Must be after the subject has been using 2 methods of birth control (as specified in Section 5.2, Inclusion Criterion #4) or complete abstinence for at least 2 months.
- Must be at least 19 days before the Day -1 pregnancy test.

9.4.2 Vital Signs

Vital signs, including heart rate, resting blood pressure, temperature, and respiratory rate, will be recorded after a 3-minute resting period according to the schedule shown in Table 3. On treatment days, vital signs should be recorded both predose and 4 hours postdose.

9.4.3 ECG

Twelve-lead ECG will be performed according to institutional standards in triplicate (3 readings in succession approximately 1 minute apart) on the visit days indicated in Table 3. The QT/QTc interval determinations from the 3 ECGs will be averaged for each time point.

9.4.4 Clinical Laboratory Tests

Table 4 identifies the clinical laboratory parameters to be measured in this study.

TABLE 4. Clinical Laboratory Tests

**Hematology**	**Serum Chemistry**	**Urinalysis**	**Other Tests**
- Hemoglobin<br>- Hematocrit<br>- White blood cell count<br>- Differential<br>- Red blood cell count<br>- Platelet count<br>- Coagulation testing: PT/INR, APTT	- Sodium<br>- Potassium<br>- Calcium<br>- BUN<br>- Creatinine<br>- Total cholesterol<br>- Total protein<br>- Albumin<br>- ALT<br>- AST<br>- Alkaline phosphatase<br>- Total bilirubin<br>- Direct bilirubin<br>- Glucose<br>- TG<br>- HDL<br>- LDL<br>- Chloride<br>- Bicarbonate<br>- Inorganic phosphate testing	- Dipstick<br>- Specific gravity<br>- pH<br>- Protein<br>- Glucose<br>- Ketones<br>- Bilirubin<br>- Blood<br>- Urobilirubin<br>- Urinalysis<br>- Microsopic urinalysis if urinalysis results are abnormal	- Thyroid function testing:<br>- free T3<br>- free T4<br>- TSH<br>- Serum retinol

Blood samples for clinical laboratory and coagulation testing will be collected after a 12-hour overnight fast on the study visit days indicated in Table 3. Urine samples will also be collected before dosing according to the schedule indicated in Table 3. (Clinical laboratory samples or urinalysis samples may also be collected on other visit days if the subject had ongoing, clinically significantly abnormal results at the preceding visit.)

Analysis of the clinical laboratory tests will be performed at a local laboratory, using the laboratory's established normal ranges and test procedures. Tests with clinically significant abnormal results must be recorded as AEs and may be repeated as necessary at the Investigator's discretion.

9.4.5 Height and Weight Measurement

Height and weight will be measured at the visits specified in Table 3. Predose height and weight measurements will be used to calculate BSA, according to the RET IRD 01 Procedure Manual.

Height will be measured at the last visit only for subjects ≤19 years of age as an assessment of bone development.

9.5 Efficacy Tests

Efficacy will be assessed based on the following tests.

9.5.1 Best-Corrected Visual Acuity: ETDRS Testing

Best-corrected visual acuity will be measured using ETDRS testing at the visits indicated in Table 3. Test distance and lighting conditions will be constant for each test. Each eye will be evaluated independently, followed by a binocular BCVA test.

See the RET IRD 01 Procedure Manual for complete instructions for BCVA using ETDRS testing.

9.5.2 Color Vision

Color vision will be tested at the visit days indicated in Table 3. Tests that may be used are Hardy-Rand-Rittler (HRR), Ishihara, or Farnsworth-Munsell 100 Hue test. At each applicable study visit, at least 1 of these 3 tests, as appropriate for the subject, should be done. More than 1 test may be done, if possible. (Farnsworth-Munsell 100 Hue test may not be appropriate for children or those with very low vision.) **For each individual subject, the test(s) done at follow-up visits must be the same as the test done at screening/baseline.** Refer to the RET IRD 01 Procedure Manual for complete instructions on conducting color vision test.

9.5.3 Visual Field Tests

Visual field tests may be done at the visit days indicated in Table 3. Because these tests can be unreliable and difficult to perform if the subject has poor or no ability to fixate, these tests will be done only if the Investigator judges the subject capable and willing to undergo the test.

All visual field tests will be performed by appropriately certified and experienced study personnel. These tests include a psycho-physical component, so testing will incorporate each subject's learning curve, and intratest variability may be assessed. The methods that may be used are Goldmann, Humphreys, or microperimetry. At each applicable study visit, at least 1 of these 3 visual field tests, as appropriate for the subject, should be done. If more than 1 test is done, they should be done in the order listed above, with microperimetry done on a separate day. **For each individual subject, the test(s) done at follow-up visits must be the same as the test done at screening/baseline.** Refer to the RET IRD 01 Procedure Manual for complete instructions for visual field tests.

9.5.4 Full-field ERG

ERG will be performed at the visit days indicated in Table 3 according to standard procedures [51]. See the RET IRD 01 Procedure Manual for complete instructions on conducting the full-field ERG.

9.6 Other Procedures

9.6.1 Blood Samples for Analysis of QLT091001 and Metabolites

Blood samples for analysis of QLT091001 and metabolites will be collected at the visits identified in Table 3. Please refer to the RET IRD 01 Study Procedure Manual for instruction on the collection, processing, storage, and shipment of samples for exploratory QLT091001, 9-cis-retinol, and other potential metabolite analysis. (Analysis is exploratory because the bioanalytical methods have not been developed and validated yet. These samples will facilitate development of the bioanalytical methods.)

9.6.2 Dynamic Pupillometry and Nystagmus Testing

Dynamic pupillometry and nystagmus testing will be performed if the appropriate instrument(s) is available at the study center. If it is available, these procedures will be performed at the visits identified in Table 3.

9.6.3 fMRI (Separate Addendum Protocol; Applies Only to Canadian Study Center)

fMRI is applicable only to the Canadian study center under a separate addendum protocol (RET IRD 01 [fMRI]). At this study center, fMRI may also be performed as long as separate informed consent is obtained. Please refer to this addendum protocol for more information.

9.6.4 Videography

Videography may be performed to document potential treatment-related changes, as equipment is available and at the discretion of the Investigator and the Sponsor.

For all videography, the identity of subjects will be kept confidential. Videography will only be performed with the consent of the subject and all study personnel who are present.

9.7 Subject Questionnaire

Subjects 12 years old or younger and/or their parent/guardian will be asked to complete selected questions (as specified on the CRF) from the Children's Visual Function Questionnaire (CVFQ). Subjects 13 years of age or older will be asked to complete selected questions (as specified on the CRF) from the Low Luminance Questionnaire (LLQ). Questionnaires will be completed at the visit days indicated in Table 3. The questionnaire should be filled out at the study center.

9.8 Instructions for Subjects

Subjects will be provided with the following instructions:

- During the first 14 days of the study. avoid exposure to direct sunlight and ultraviolet (UV) light, or use a sunscreen with SPF 15 or greater and wear sunglasses.
- During the first 14 days of the study, avoid new nonprescription medications, vitamin and dietary supplements (particularly vitamin A), alcohol, and drugs of abuse (subjects should be advised that random drug and alcohol testing may be performed). If any new medication is started during the study, inform the Investigator immediately.
- Avoid vigorous physical activity such as running, sports activities, dancing, or gym workouts. Physical activity should be limited starting from 3 days prior to dosing until Day 14/15 of the study.
- Do not schedule elective surgery from 14 days prior to dosing until after Day 30 of the study.
- Follow the dietary guidelines as described below:
    - No coffee, tea, or other beverages with caffeine (such as cola) from Day 0 until after Day 14/15 study procedures have been completed.
    - Consumption of alcohol is prohibited until after Day 14/15. Subsequently during the follow-up period, alcohol intake should be limited to no more than 1 drink/day with meals, and alcohol should not be consumed on the day of study assessments.
    - The following foods should be avoided during the study, from 12 hours before the first screening blood test until after the Day 14/15 visit.

      Avoid:
      liver (any kind)
      energy drinks and energy bars

vitamin drinks and shakes, such as Vitaminwater, Ovaltine™ & Milo™
butter
carrots
mangos
papaya
kale
spinach
dried apricots or apricot juice
canned soup (ready to serve)

Limit to a maximum of 1 serving per day:

cantaloupe
milk (regular and powdered milk)
cheese
egg yolk (to a maximum of 2 per week)
sweet potatoes
margarine

- Avoid pregnancy / fathering a child during the treatment phase of the study and for 2 months after finishing the study drug by:
  - Men and boys: abstaining completely from sexual intercourse, vasectomy, or using a barrier method (condoms) with spermicide during sexual intercourse
  - Women and girls: abstaining completely from sexual intercourse or using methods of birth control as specified in Section 5.2, Inclusion Criterion #3.

10 EVALUATION, RECORDING, AND REPORTING OF ADVERSE EVENTS

All adverse events (AEs) either observed by the Investigator or one of his/her medical collaborators, or reported by the subject spontaneously, or in response to the direct question below, will be noted in the AEs section of the subject's CRF and in the source document. Only treatment-emergent AEs (those occurring during or after the start of study treatment) should be recorded as AEs. Events reported before the initial study treatment should be recorded as medical history.

In an attempt to optimize consistency of AE reporting across centers, the subject must be asked a standard question to elicit any AEs. At each clinic or telephone evaluation of the subject, study personnel will ask the following question: "Have you had any problems since your last visit or telephone call?"

If any AE is reported, the date of onset, intensity, relationship to study medication or treatment, date of resolution (or the fact that it is still continuing or has become chronic), and whether the AE is serious or not will be recorded (see Section 10.2).

For any change in laboratory results, vital signs, or ECG measurements that arises after treatment, the Investigator will decide if the value is clinically significant. The Investigator

will determine if it is necessary to repeat the evaluation. If the evaluation is judged to be clinically significant, it must be recorded as an AE.

10.1 Definitions

10.1.1 Adverse Event (AE)

Adverse Event (AE): any unfavorable and unintended sign (including a clinically significant abnormal laboratory finding), symptom, or disease temporally associated with the administration of a medicinal product, whether or not considered related to the investigational product or device.

Medical conditions or diseases present before a subject starts study treatment are only considered AEs if they worsen after the subject starts study treatment.

10.1.2 Serious Adverse Events (SAEs)

Serious Adverse Event (SAE): defined as any AE that (at any dose):

- Results in death
- Is life-threatening

  The term "life-threatening" refers to an event in which the subject was at risk of death at the time of the event; it does not refer to an event which hypothetically might have caused death if it were more intense.

- Requires inpatient hospitalization or prolongs existing hospitalization
- Results in persistent or significant disability / incapacity
- Is a congenital anomaly / birth defect
- May jeopardize the subject or may require intervention to prevent one of the outcomes listed above. Medical and scientific judgment should be exercised in deciding if these events should be considered serious. Examples of such events are intensive treatment in an emergency room or at home for allergic bronchospasm; blood dyscrasias or convulsions that do not result in hospitalization; or development of drug dependency or drug abuse.

A subject admitted to a hospital as a result of an AE, even if released on the same day, would qualify for inpatient hospitalization. An emergency room visit that results in admission to the hospital would also qualify for inpatient hospitalization. However, emergency room visits that do not result in admission to the hospital would not qualify for inpatient hospitalization and, instead, should be evaluated for one of the other criteria for SAEs (e.g., life-threatening AE or medically significant event).

Hospitalization scheduled before a subject enrolls in the study is not the result of a treatment-emergent AE, and therefore events leading to such hospitalization will not be considered

study AEs or SAEs. During the study, if a subject has elective surgery for a condition present at inclusion into the study, and the condition did not worsen during the study, the reason for elective surgery (and resulting hospitalization, if applicable) should not be considered or reported as an SAE. (Surgery or hospitalization should always be reported as an outcome of an AE.) For AEs that result in persistent or significant disability/incapacity, disability/incapacity refers to a substantial disruption of a subject's ability to carry out normal life functions.

10.2 Adverse Event Descriptions

10.2.1 Intensity

The intensity of AEs will be characterized as mild, moderate, or severe, as follows:

Mild	Usually transient, requiring no special treatment, and does not interfere with the subject's daily activities
Moderate	Introduces a low level of inconvenience or concern to the subject and may interfere with daily activities, but is usually ameliorated by simple therapeutic measures
Severe	Significantly interferes with a subject's usual daily activities and requires systemic drug therapy or other treatment, if available.

10.2.2 Relationship to Study Treatment

The causal relationship to study drug or treatment will be determined by the Investigator according to best medical judgment, as follows:

Suspected	There is a reasonable possibility that the AE is associated with use of the study treatment, such as a temporal relationship of the event to study treatment administration, or when other drugs, therapeutic interventions, or underlying conditions do not provide a sufficient explanation for the observed event.
Not suspected	A relationship between the AE and the study treatment can reasonably be ruled out based on lack of any temporal relationship of the event to study treatment administration, or when the subject's underlying condition, medical history, or other therapy provide sufficient explanation for the observed event.

10.3 Reporting and Evaluation of Serious Adverse Events and Other Clinically Significant Adverse Events

Any SAE occurring in this study must be reported immediately (within 24 hours) by email to the Sponsor safety designated contact listed below.

< *contact identity redacted* >

Other procedural and contact details on SAE reporting to < *contact identity redacted* > (including country-specific numbers of toll-free fax lines) will be available in the Investigator Site File.

In case of emergency or a need for urgent contact, Investigators should contact the Medical Monitor:

< *contact identity redacted* >

The Investigator must also submit documentation of the following to the Institutional Review Board, Ethics Committee, or Research Ethics Board (IRB):

- Center-specific SAEs and follow-up to these SAEs: The type of SAE that must be submitted (e.g., all SAEs or only suspected SAEs), as well as the required timing of submission (e.g., within 15 days of occurrence), is defined by the IRB or regulatory authorities.
- All reportable SAEs from the study: The Sponsor's safety designee will provide documentation of reportable SAEs to the Investigator, as specified in Section 14.1.3.

All Suspected Unexpected Serious Adverse Reactions (SUSARS) will be reported to the local Regulatory Authorities, Institutional Review Board, Ethics Committee, or Research Ethics Board (IRB) by the Sponsor's safety designee.

The Investigator should ensure that the subject receives appropriate medical treatment and that the subject is followed up until the SAE resolves or becomes chronic, as defined in Section 10.5.

10.4 Adverse Event Definitions for Discontinuation Criteria

If the subject is discontinued due to the hematology, transaminase, serum lipid, thyroid parameter, serum retinol, ECG parameter, hypersensitivity to study treatment, or clinical signs of hypervitaminosis A criteria specified in Section 5.4, the reason for withdrawal will be recorded as an AE, and the subject will be withdrawn from study treatment due to this AE.

10.5 Follow-up for Adverse Events

Throughout the study to the final study visit, all AEs will be followed until they resolve or become chronic (as judged by the Investigator).

At the final study visit, new AEs, as well as follow-up information for continuing AEs, will be recorded in the CRF and source document. If an SAE (defined in Section 10.1.2) is unresolved at the final study visit, it will be followed by the Investigator until it resolves or becomes chronic (as judged by the Investigator). Follow-up data for such SAEs will be recorded in the source document and reported to the safety contacts (refer to Section 10.3). Non-serious ongoing AEs will be followed beyond the final study visit at the discretion of the Investigator and recorded in the source documents.

10.6 Pregnancy Follow-up

If a subject becomes pregnant during the study, the Investigator must inform the Sponsor and collect follow-up data regarding the pregnancy, birth, and status of the child. The Sponsor will provide special CRFs for data collection in the case of pregnancy. Follow-up should be continued until study close-out at the study center. After close-out, QLT Safety will continue to obtain follow-up information.

Pregnancy should be recorded as a protocol deviation. Pregnancy is not an AE; however, any complication related to pregnancy would be considered an AE.

10.7 Reporting of Technical Complaints about the Investigational Drug

10.7.1 Definitions

Drug Technical Complaint: A quality complaint received in writing, electronically, or orally that involves the use or attempted use of a drug product that identified any defects in the physical properties of the drug product (color, precipitates, viscosity, etc.) or its packaging. Drug technical complaints also include any identified customer dissatisfaction with the physical characteristic(s) of the drug product (dispensing characteristics, labeling, packaging, etc.).

10.7.2 Reporting of Technical Complaints

Any technical complaint should be reported by fax to the Sponsor's Quality Assurance Complaint Coordinator (contact information below) within 24 hours. The complaint report should include the following information:

- Name of the drug.
- Strength of drug.
- Batch/lot number on the drug container.
- Investigator name, study center name, and contact number.
- Date the complaint occurred.
- Brief description of the complaint.

- Subject involved (yes or no); if yes, were any AEs associated with the complaint (yes or no). (If a subject AE is associated with the complaint, refer to Section 10.)

The drug container which initiated the complaint should be returned to the Quality Assurance Complaint Coordinator (address below) for analysis.

QLT Inc.
Attn: Quality Assurance Complaint Coordinator
887 Great Northern Way, Suite 101
Vancouver, B.C.
Canada
V5T 4T5
< contact information redacted >

Any complaint about a drug product or device must be reported regardless of whether the defect or deficiency had any effect on a subject or on study personnel.

11 STATISTICAL CONSIDERATIONS

11.1 Sample Size

The sample size of 28 subjects with LCA or RP caused by *RPE65* or *LRAT* mutation (up to 14 subjects for each disease cohort) will result in 7 subjects per genotype subgroup per disease cohort. This number is based on the small population of these LCA and RP genotypes and on clinical judgment that this is a sufficient number of subjects to meet the objectives of the study.

11.2 Data Sets to be Analyzed

11.2.1 Efficacy: Intent to Treat

The intent-to-treat (ITT) data set will include data from all enrolled subjects. All data will be included and no subjects will be excluded because of protocol violations.

11.2.2 Safety

The safety data set will include data from all enrolled subjects who receive any treatment. In the safety analysis, no data exclusion is allowed because of protocol violations.

11.3 Analysis of Demographic and Baseline Data

The demographic and baseline analyses will be done for the ITT data set.

Subject demographic and baseline characteristics will be summarized by gene mutation subgroup and disease cohort with mean, standard deviation, median, minimum, and maximum for continuous variables; and by counts and percentages for categorical variables.

11.4 Efficacy Variables and Analyses

Efficacy analyses will be performed on data from both eyes of all subjects included in the ITT data set. Each eye will be evaluated separately.

11.4.1 Best-Corrected Visual Acuity Using ETDRS Testing

Visual acuity scores based on ETDRS testing and changes from baseline will be provided for each time point by gene mutation subgroup and disease cohort using descriptive statistics (mean, standard deviation, minimum, median, and maximum).

11.4.2 ERG Variables

Analysis of the ERG will include variables of peak time and amplitude. The a-wave amplitude will be measured from baseline to trough and b-wave amplitude will be measured from a-wave trough to b-wave peak. Peak times will be measured from the flash onset to the peak of the wave under evaluation.

The responses of both eyes for each stimulus condition will be evaluated separately, and a- and b-wave amplitudes and implicit times will be measured in photopic and scotopic conditions to estimate the cone and rod contributions, respectively. B-wave amplitudes will then be plotted against the corresponding flash intensity in order to generate the photopic and scotopic luminance-response functions from which the rod and cone V_{max}, retinal sensitivity (log K) and *ka* and *kd* (for the *Photopic Hill*) parameters can be calculated according to previously reported methods.

ERG variables and changes from baseline will be summarized by gene mutation subgroup and disease cohort using descriptive statistics (mean, standard deviation, minimum, median, and maximum). In addition, the a-wave and b-wave amplitudes will be presented graphically for each group by flash intensity.

11.4.3 Goldmann Visual Field (GVF)

GVF results [52] will be interpreted and quantified independently by researchers at Johns Hopkins University, using a method described in Dagnelie [53] and used extensively in RP. The observed GVF and log transformation of GVF, as well as their respective change and percent change from baseline for the large (V4e) and smaller (III4e and I4e) targets will be summarized for all subjects using descriptive statistics (mean, standard deviation, minimum, median, and maximum).

11.4.4 Other Efficacy Variables

Other variables (visual field [other than GVF], color vision, dynamic pupillometry, nystagmus testing, OCT, and FAF) will be summarized by gene mutation subgroup, disease

cohort, and visit using mean, standard deviation, median, minimum, and maximum for continuous variables; and counts and percentages for categorical variables.

11.5 Safety Variables and Analyses

Safety analyses will be performed on all subjects who received treatment (safety data set). This study has the following safety variables:

- Adverse events (AEs)
- Concomitant medications
- Vital signs (temperature, blood pressure, heart rate, respiratory rate)
- Clinical laboratory tests (including hematology, chemistry, urinalysis, thyroid function, serum retinol, and coagulation testing)
- Electrocardiogram (ECG) measurements
- Height
- Biomicroscopy, IOP, and dilated fundus exam
- BCVA score

AEs will be coded using the Medical Dictionary for Regulatory Activities (MedDRA) with the number and percentage of subjects experiencing an AE and the total number of events summarized with system organ class and preferred term. Concomitant medications will be coded with the World Health Organization Drug Dictionary (WHO DD) and tabulated. Other safety variables including clinical laboratory test results and vital signs will be summarized with appropriate descriptive statistics. ECG, biomicroscopy, IOP, dilated fundus exam, and cycloplegic refraction results will be listed by subject. See Section 9.5 for details on the visual acuity score.

11.6 Other Analyses

11.6.1 Subject Questionnaire

The subject questionnaire will be summarized with appropriate descriptive statistics by gene mutation subgroup and disease cohort.

11.6.2 QLT091001 Levels

Serum levels of QLT091001 and any metabolites, if available, will be listed by subject, and may be summarized with appropriate descriptive statistics if data permit.

12 ESTIMATED DURATION OF THE STUDY

This study started in the fall of 2009 and is expected to be completed in the spring of 2013.

13 STUDY ETHICAL CONSIDERATIONS

13.1 Ethical Conduct of the Study

The study will be conducted in accordance with the ICH E6: Good Clinical Practice: Consolidated Guideline, and constituted in keeping with the principles of ICH E8: General Considerations for Clinical Trials; Part C, Division 5 of the Canadian Food and Drug Regulations; and US 21 CFR Parts 50, 54, and 56; as applicable.

13.2 Informed Consent and Assent

The informed consent forms used for the study must comply with the applicable laws and regulations ICH E6: Good Clinical Practice: Consolidated Guideline, and must have been approved by the Sponsor (prior to review by the IRB). Any subsequent changes required by the IRB must also be approved by the Sponsor. An Investigator must explain the medical aspects of the study, including the nature of the study and the treatment, orally and in writing, in such a manner that the subject and the subject's parent or legal guardian are aware of potential benefits and risks. Other elements of the informed consent process may be delegated by the Investigator. Subjects and the subject's parent or legal guardian must be informed about all aspects of the clinical trial that are necessary to make the decision to participate in the clinical trial. Subjects and the subject's parent or legal guardian must be informed that participation is voluntary and that they may withdraw from the study at any time, without prejudice. Documentation of the discussion and the date of informed consent must be recorded in the source documentation. The subject's parent or legal guardian must give informed consent in writing.

Even though the study will be enrolling subjects who are not legally able to provide consent for themselves, the assent of study subjects 7 years and older must also be solicited, according to the guidelines of the study center's IRB.

The informed consent process must be conducted, and the form must be signed, before the subject undergoes any Screening procedures that are performed solely for the purpose of determining eligibility for the study.

13.3 Institutional Review Board, Ethics Committee, or Research Ethics Board (IRB)

The protocol, protocol amendments (as specified by the IRB), and the informed consent form for the proposed study, along with any other documents required by the center's IRB must be submitted by the Investigator to the center's duly constituted IRB for review and approval. The Investigator must also ensure that the IRB reviews the progress of the study on a regular basis and, if necessary, renews its approval of the study on an annual basis. A copy of each IRB approval letter must be forwarded to the Sponsor and/or their representatives before the study is implemented. Documentation of subsequent reviews of the study must also be forwarded to the Sponsor and/or their representatives.

14 ADMINISTRATIVE PROCEDURES

14.1 Sponsor's Responsibilities

The following are responsibilities of the Sponsor and/or their representatives.

14.1.1 Study Supplies

The Sponsor will supply sufficient quantities of the following materials to the clinical center:

- Study drug as described in Section 7
- Investigator's Brochure (IB) for study drug
- Any forms and logs required for study completion

14.1.2 Investigator Training

The study center will have a center-specific study initiation meeting to ensure the center staff understands the protocol, study requirements, and data capture processes. This training will take place prior to enrollment of the first subject at each study center. Each study center will be provided with information regarding GCP and regulations specific to the conduct of clinical studies.

14.1.3 Ongoing Communication of Safety Information During the Study

The Sponsor and/or their representatives will provide the Investigator with documentation of SAEs reported to regulatory authorities (reportable SAEs) during the conduct of the study. The Investigator must forward this documentation to the IRB, as described in Section 10.3.

The Sponsor and/or their representatives will also notify the Investigator about any other safety findings that could affect the safety of subjects, affect the conduct of the study, or alter the IRB's opinion about continuation of the study.

14.1.4 Study Monitoring

The study will be monitored by representatives of QLT. Routine monitoring visits will be conducted to:

- Ensure compliance with the study protocol.
- Verify that the informed consent process was conducted before initiation of any Screening procedures that are performed solely for the purpose of determining eligibility for the study and prior to the provision of study medication.
- Verify that the protocol, protocol amendments, and safety information are submitted to the IRBs in a timely manner.
- Review the CRFs and source documents to ensure that reported study data are accurate, complete, and verifiable from source documents.

- Verify that the investigational products are stored properly and under the proper conditions, that they are in sufficient supply, and that receipt, use, and return of investigational products at the study centers are controlled and documented adequately.
- Verify that the Investigator and study center personnel remain adequately qualified throughout the study.
- Verify that the research facilities, including laboratories and equipment, are maintained adequately to safely and properly conduct the study.

14.1.5 Records Retention

The Sponsor must retain all documentation pertaining to the study according to QLT standard operating procedures.

14.2 Investigator's Responsibilities

14.2.1 Reporting and Recording of Study Data

Data will be captured and compiled using procedures developed by the Sponsor or their representatives. All requested study data must be recorded clearly on the Case Report Form (CRF) and other study forms as required. An explanation should be provided for all missing data. Only individuals who are identified on the Study Personnel Identification List may enter or correct data in the CRF. Incomplete or inconsistent data on the CRFs will result in data queries that require resolution by the Investigator.

The protocol, informed consent form, protocol amendments, safety information, and other required documents must be submitted to the IRB in a timely manner, as described in Section 10.3 (for safety information) and Section 13.3 (for other documents).

14.2.2 Source Documentation

The Investigator must maintain adequate and accurate source documents upon which CRFs for each subject are based. They are to be separate and distinct from CRFs, except for cases in which the Sponsor has predetermined that direct data entry into specified pages of the subject's CRF is appropriate. These records should include detailed notes on:

- The oral and written communication with the subject regarding the study treatment (including the risks and benefits of the study). The date of informed consent must be recorded in the source documentation.
- The subject's medical history prior to participation in the study.
- The subject's basic identifying information, such as demographics, that links the subject's source documents with the CRFs.
- The results of all diagnostic tests performed, diagnoses made, therapy provided, and any other data on the condition of the subject.
- The subject's exposure to study treatment.

- All AEs.
- The subject's exposure to any concomitant therapy (including start and stop dates, route of administration, and dosage).
- All relevant observations and data on the condition of the subject throughout the study.

14.2.3 Study Drugs

The Investigator is responsible for ensuring the study drugs are administered or dispensed only to subjects enrolled in the study. An accurate accounting of the study drugs must be maintained using a separate form. These records must show dates, lot numbers, and quantities received, dispensed, and returned. The Investigator will ensure that any used and unused vials of study drug and other study material will be returned to the Sponsor on completion of the study.

14.2.4 Records Retention

The Investigator must ensure that clinical study records are retained according to the applicable country regulations, as documented in the clinical trial agreement entered into with the Sponsor or Sponsor representatives in connection with this study.

Clinical study records, subject files and other source data must be kept for the maximum period of time required by applicable country regulations or required by the hospital, institution, or private practice (whichever is longest). The Investigator must inform the Sponsor immediately if any documents are to be destroyed, to be transferred to a different facility, or to be transferred to a different owner.

15 POLICY FOR PUBLICATION AND PRESENTATION OF DATA

< *Sponsor policy redacted* >

16 REFERENCES

1. Marcus R, Coulston AM. Fat soluble vitamins. In: Hardman JG, Limbird LE, Molinoff PB, Ruddon RW, Gilman AG, eds. Goodman & Gilman's The Pharmacological Basis of Therapeutics. 9th ed. 1996:1573-1590.

2. Accutane® (isotretinoin) US prescribing information. Available at: http://dailymed.nlm.nih.gov/dailymed/fda/fdaDrugXsl.cfm?id=1259&type=display. Accessed 23 October 2007.

3. Accutane® (isotretinoin) Canadian product monograph. Available at: http://cpe0013211b4c6d-cm0014e88ee7a4.cpe.net.cable.rogers.com/dpdonline /displayInfo.do? drugCode=ovMYdjKz7EU%3D. Accessed 3 March 2008.

4. Soriatane® (acitretin) US prescribing information. Available at: http://dailymed.nlm.nih.gov/dailymed/fda/fdaDrugXsl.cfm?id=2753&type=display. Accessed 23 October 2007.

5. Soriatane® (acitretin) Canadian product monograph. Available at: http://cpe0013211b4c6d-cm0014e88ee7a4.cpe.net.cable.rogers.com/dpdonline/ displayInfo.do?drugCode=PdLVhg%2BtG2c%3D. Accessed 3 March 2008.

6. Vesanoid® (tretinoin) capsules US Prescribing information. Available at: http://dailymed.nlm.nih.gov/dailymed/fda/fdaDrugXsl.cfm?id=696&type=display. Accessed 23 October 2007.

7. Vesanoid® (all-trans-retinoic acid / tretinoin) Canadian product monograph. Available at: http://cpe0013211b4c6d-cm0014e88ee7a4.cpe.net.cable.rogers.com/ dpdonline/ searchRequest.do. Accessed 3 March 2008.

8. Targretin® (bexarotene) capsules US Prescribing information. Available at: http://dailymed.nlm.nih.gov/dailymed/fda/fdaDrugXsl.cfm?id=493&type=display. Accessed 23 October 2007.

9. Kuksa V, Imanishi Y, Batten M, Palczewski K, and Moise AR. Retinoid cycle in the vertebrate retina: experimental approaches and mechanisms of isomerization. Vis Res. 2003;43:2959-2981.

10. Van Hooser JP, Aleman TS, He Y-G, et al. Rapid restoration of visual pigment and function with oral retinoid in a mouse model of childhood blindness. Proc Natl Acad Sci 2000; 97(15):8623-8628.

11. Bainbridge JWB, Barker SS, Henderson R, et al. Effect of gene therapy on visual function in Leber's congenital amaurosis. New Engl J Med. 2008;358:2231-2239.

12. Entrez Gene: LRAT lecithin retinol acyltransferase (phosphatidylcholine--retinol O-acyltransferase). Available at: http://www.ncbi.nlm.nih.gov/sites/entrez?Db=gene&Cmd=ShowDetailView&TermToSearch=9227 Accessed on 19 December 2008.

13. Biesalski HK and Nohr D. New aspects of vitamin A metabolism: the role of retinyl esters as systemic and local sources for retinol in mucous epithelia. J Nutr. 2004;134:3453S-3457S.

14. Mata NL, Moghrabi WN, Lee JS. RPE65 is a retinyl ester binding protein that presents insoluble substrate to the isomerase in retinal pigment epithelial cells. J Biol Chem. 2004;279(1):635-643.

15. Redmond TM, Poliakov E, Yu S, Tsai JY, Lu Z, Gentleman S. Mutation of key residues of RPE65 abolishes its enzymatic role as isomerohydrolase in the visual cycle. Proc Natl Acad Sci USA. 2005;102(38):13658-13663.

16. Jin M, Li S, Moghrabi WN, Sun H, Travis GH. Rpe65 is the retinoid isomerase in bovine retinal pigment epithelium. Cell. 2005;122:449-459.

17. Moiseyev G, Chen Y, Takahashi Y, Ma JX. RPE65 is the isomerohydrolase in the retinoid visual cycle. Proc Natl Acad Sci USA. 2005;102(35):12413-12418.

18. Koenekoop RK, Loyer M, Dembinska O, Beneish R. Visual improvement in Leber congenital amaurosis and the CRX genotype. Ophthalmic Genet. 2002;23(1):49-59.

19. den Hollander AI, Roepman R, Koenekoop RK, Cremers FP. Leber congenital amaurosis: genes, proteins and disease mechanisms. Prog Retin Eye Res. 2008;27(4):391-419.

20. RetNet – Retinal Information Network. The University of Texas-Houston Health Science Center. Available at: http://www.sph.uth.tmc.edu/Retnet/. Accessed on 17 December 2008.

21. Perrault I, Delphin N, Hanein S, et al. Spectrum of NPHP6/CEP290 mutations in Leber congenital amaurosis and delineation of the associated phenotype. Hum Mutat. 2007;28(4):416.

22. Phelan JK, Bok D. A brief review of retinitis pigmentosa and the identified retinitis pigmentosa genes. Mol Vis. 2000:6:116-124.

23. Van Hooser JP, Liang Y, Maeda T, et al. Recovery of visual functions in a mouse model of Leber congenital amaurosis. J Biol Chem. 2002;277(21):19173-19182.

24. Maeda A, Maeda T, Golczak M, et al. Effects of potent inhibitors of the retinoid cycle on visual function and photoreceptor protection from light damage in mice. Mol Pharmacol. 2006;70:1220-1229.

25. Kandori H, Maeda A. FTIR spectroscopy reveals microscopic structural changes of the protein around the rhodopsin chromophore upon photoisomerization. Biochemistry, 1995;34:14220-14229.

26 Second Sight Medical Products, Inc. Second Sight Medical Products Announces European Market Approval of a Retinal Prosthesis for the Blind. Press release. 2 Mar 2011. Available at: http://www.2-sight.eu/en/press-releases-en/press-releases-2011-en. Accessed on 19 September 2011.

27. Maguire AM, Simonelli F, Pierce EA, et al. Safety and efficacy of gene transfer for Leber's Congenital Amaurosis. New Engl J Med. 2008;358:2240-2248.

28. Cideciyan AV, Aleman TS, Boye SL, et al. Human gene therapy for RPE65-isomerase deficiency activates the retinoid cycle of vision but with slow rod kinetics. Proc Natl Acad Sci USA. 2008;105(39):15112-15117.

29. Hauswirth WW, Aleman TS, Kaushal S, et al. Treatment of Leber congenital amaurosis due to RPE65 mutations by ocular subretinal injection of adeno-associated virus gene vector: short-term results or a phase I trial. Hum Gene Ther. 2008;19(10):979-990. Available online 7 Sept 2008.

30. Koenekoop RK. Successful PRE65 gene replacement and improved visual function in humans. Ophthalmic Genet. 2008;29:89-91.

31. < *Sponsor internal report redacted* >

32. < *Sponsor internal report redacted* >

33. < *Sponsor internal report redacted* >

34. < *Sponsor internal report redacted* >

35. < *Sponsor internal report redacted* >

36. < *Sponsor internal report redacted* >

37. < *Sponsor internal report redacted* >

38. < *Sponsor internal report redacted* >

39. Silverman AK, Ellis CN, Voorhees JJ. Hypervitaminosis A syndrome: a paradigm of retinoid side effects. J Am Acad Dermatol. 1987;16(5 Pt 1):1027-1039.

40. < *Sponsor internal report redacted* >

41. < *Sponsor internal report redacted* >

42. < *Sponsor internal report redacted* >

43. Weleber RG, Francis PJ, Trzupek KM. Leber congenital amaurosis. Available at: http://www.ncbi.nlm.nih.gov/bookshelf/br.fcgi?book=gene&part=lca. Accessed on 8 May 2009.

44. Hartong DT, Berson EL, Dryja TP. Retinitis pigmentosa. Lancet. 2006;368:1795-1809.

45. Maguire AM, High KA, Auricchio A, et al. Age-dependent effects of rpe65 gene therapy for leber's congenital amaurosis: a phase 1 dose-escalation trial. Lancet. 2009;374:1597-1605.

46. O'Byrne SM, Wongsiriroj N, Libien J, Vogel S, et al. Retinoid absorption and storage is impaired in mice lacking lecithin:retinol acyltransferase (LRAT). J Biol Chem. 2005;280(42):35647-35657. Epub 2005 Aug 22.

47. Batten ML, Imanishi Y, Maeda T, Tu DC, et al. Lecithin-retinol acyltransferase is essential for accumulation of all-trans-retinyl esters in the eye and in the liver. J Biol Chem. 2004;279(11):10422-10432. Epub 2003 Dec 18.

48. Koenekoop RK, Racine J, Al Humaid S, et al. Oral Synthetic cis-Retinoid Therapy in Subjects with Leber Congenital Amaurosis (LCA) due to Lecithin: Retinol Acyltransferase (LRAT) or Retinal Pigment Epithelial 65 Protein (RPE65) mutations: Preliminary Results of a Phase Ib Open Label Trial. ARVO presentation 3323/A571; 3 May 2011. Available at: http://www.abstractsonline.com/Plan/ViewAbstract.aspx?sKey=9456c86d-1d58-492c-ab4f-bc1dfbebebee&cKey=c1849603-6a33-4902-b43d-cdcc29d30c6d&mKey=%7b6F224A2D-AF6A-4533-8BBB-6A8D7B26EDB3%7d. Accessed on 9 April 2011.

49. Institute of Medicine. Food and Nutrition Board. Dietary Reference Intakes for Vitamin A, Vitamin K, Arsenic, Boron, Chromium, Copper, Iodine, Iron, Manganese, Molybdenum, Nickel, Silicon, Vanadium, and Zinc. National Academy Press, Washington, DC, 2001. Available at: http://www.nutrisci.wisc.edu/NS623/drivitasum.pdf. Accessed on 8 May 2009.

50. < *Sponsor internal report redacted* >

51. Marmor MF, Holder GE, Seeliger MW, Yamamoto S. Standard for clinical electroretinography (2004 update). Doc Ophthalmol. 2004;108:107-114.

52. Berson EL, Rosner B, Sandberg MA, et al. A randomized trial of vitamin A and vitamin E supplementation for retinitis pigmentosa. Arch Ophthalmol. 1993;111:761-772.

53. Dagnelie G. Conversion of planimetric visual field data into solid angles and retinal areas. Clinical Vision Sciences. 1990;5:95–100.
