# Supplementary material for: Safety and Proof-of-Concept Study of Oral QLT091001 in Retinitis Pigmentosa Due to Inherited Deficiencies of Retinal Pigment Epithelial 65 Protein (RPE65) or Lecithin:Retinol Acyltransferase (LRAT)
Source: PLoS One. 2015 Dec 10;10(12):e0143846. doi: 10.1371/journal.pone.0143846 (PMC4687523; doi:10.1371/journal.pone.0143846)
Supplement: S1 Table — (PDF) [file pone.0143846.s008.pdf]

S1 Table. Demographics.

Characteristics				
Age (y)	n		18	
	Mean		28.54	
	SD		11.46	
	Min.		6.4	
	Max.		55.9	
Age Categories in years	6-11	n	2	(11%)
	12-17	n	0	
	18-65	n	16	(89%)
Gender	n		18	
	Male		10	(56%)
	Female		8	(44%)
Race	n		18	
	Caucasian		11	(61%)
	Black		0	
	Asian		6	(33%)
	Hispanic		1	(6%)
	Other		0	
Gene Deficiency	n		18	
	LRAT		5	(28%)
	RPE65		13	(72%)
Height (cm)	n		18	
	Mean		169.6	
	SD		15.9	
	Min.		122	
	Max.		193	
Weight (kg)	n		18	
	Mean		71.01	
	SD		23.03	
	Min.		22.8	
	Max.		117.6	
BSA (m^2)	n		18	
	Mean		1.814	
	SD		0.372	
	Min.		0.88	
	Max.		2.42	
GVF log retinal area	n		18	

Characteristics		
(mm^2) for primary isopter (OD)	Mean	1.834
	SD	0.569
	Median	2.045
	Min.	0.48
	Max.	2.53
GVF log retinal area (mm^2) for primary isopter (OS)	n	18
	Mean	1.752
	SD	0.600
	Median	1.820
	Min.	0.40
	Max.	2.53
VA (letter score) (OD)	n	18
	Mean	30.4
	SD	24.0
	Median	28.0
	Min.	0
	Max.	71
VA (letter score) (OS)	n	18
	Mean	30.4
	SD	21.0
	Median	28.5
	Min.	0
	Max.	62
