# Supplementary material for: Safety and Proof-of-Concept Study of Oral QLT091001 in Retinitis Pigmentosa Due to Inherited Deficiencies of Retinal Pigment Epithelial 65 Protein (RPE65) or Lecithin:Retinol Acyltransferase (LRAT)
Source: PLoS One. 2015 Dec 10;10(12):e0143846. doi: 10.1371/journal.pone.0143846 (PMC4687523; doi:10.1371/journal.pone.0143846)
Supplement: S2 Table — (PDF) [file pone.0143846.s009.pdf]

S2 Table. Summary of All Adverse Drug Reactions.

System Organ Class:<br>*- Event*	*# and %*<br>*of Patients*		*# of*<br>*Events*
Any adverse drug reaction	18	(100%)	141
Eye Disorders:			
- Any event	7	(39%)	21
- Erythema of eyelid	1	(6%)	1
- Glare	1	(6%)	2
- Photophobia	5	(28%)	10
- Photopsia	2	(11%)	8
Gastrointestinal Disorders:			
- Any event	5	(28%)	10
- Abdominal pain	1	(6%)	1
- Nausea	3	(17%)	6
- Vomiting	2	(11%)	3
General Disorders:			
- Any event	4	(22%)	8
- Fatigue	2	(11%)	3
- Feeling hot	1	(6%)	2
- Orthostatic intolerance	1	(6%)	1
- Pyrexia	2	(11%)	2
Investigations:			
- Any events	9	(50%)	21
- Alanine aminotransferase increased	4	(22%)	4
- Aspartate aminotransferase increased	6	(33%)	6
- Blood cholesterol increased	1	(6%)	1
- Blood potassium increased	1	(6%)	1
- Blood triglycerides increased	2	(11%)	2
- Hematocrit decreased	1	(6%)	1
- Hemoglobin decreased	1	(6%)	1
- High density lipoprotein decreased	3	(17%)	3
- Low density lipoprotein increased	1	(6%)	1
- Thyroxine free decreased	1	(6%)	1
Metabolism and Nutrition Disorders:			
- Any event	1	(6%)	1
- Decreased appetite	1	(6%)	1
Nervous System Disorders:			
- Any event	17	(95%)	73
- Dysesthesia	1	(6%)	1
- Headache	17	(95%)	69
- Somnolence	2	(11%)	2
- Vertigo	1	(6%)	1

System Organ Class:<br>*- Event*	*# and %*<br>*of Patients*		*# of*<br>*Events*
Skin and Subcutaneous Tissue Disorders:			
- Any event	2	(11%)	2
- Erythema	1	(6%)	1
- Pruritus	1	(6%)	1
Vascular Disorders:			
- Any event	2	(11%)	5
- Flushing	1	(6%)	3
- Hot flush	1	(6%)	2
