# Supplementary material for: Safety and Proof-of-Concept Study of Oral QLT091001 in Retinitis Pigmentosa Due to Inherited Deficiencies of Retinal Pigment Epithelial 65 Protein (RPE65) or Lecithin:Retinol Acyltransferase (LRAT)
Source: PLoS One. 2015 Dec 10;10(12):e0143846. doi: 10.1371/journal.pone.0143846 (PMC4687523; doi:10.1371/journal.pone.0143846)
Supplement: S1 Text — (PDF) [file pone.0143846.s010.pdf]

S1 Text. Visit Schedule.

The study was originally planned to continue at least through 12 months with follow up visits at approximately days 7/8, 14/15 and 30; and months 2, 4, 6, 8, 10, and 12. Following a protocol amendment, patients could have completed the study at the Day 30 visit, or at any visit after that, if they met entry criteria and were enrolled into a subsequent study for retreatment with QLT091001 (RET IRD 02 trial; ClinicalTrials.gov Identifier: NCT01521793). Because those patients were enrolled into the re-retreatment trial (which, by definitions of the Food and Drug Administration, was a different trial) they were retracted from the RET IRD 01 trial and enrolled into the RET IRD 02 trial. All patients had at least 2 months of follow-up and 13 RP patients (72%) had 8 months of follow-up for at least one of GVF or VA. There were no visits for RP patients beyond Month 8.
