# Supplementary material for: Safety and Proof-of-Concept Study of Oral QLT091001 in Retinitis Pigmentosa Due to Inherited Deficiencies of Retinal Pigment Epithelial 65 Protein (RPE65) or Lecithin:Retinol Acyltransferase (LRAT)
Source: PLoS One. 2015 Dec 10;10(12):e0143846. doi: 10.1371/journal.pone.0143846 (PMC4687523; doi:10.1371/journal.pone.0143846)
Supplement: S2 Text — (PDF) [file pone.0143846.s011.pdf]

Supplementary material 2: Exclusion criteria

Patients were excluded from the study if they had taken any oral retinoid medication in the past 6 months or had shown at any time intolerance to previous retinoid medication, had clinically important abnormal physical findings at screening, had a history of diabetes or chronic hyperlipidemia, hepatitis, pancreatitis or cirrhosis, liver failure, uncontrolled thyroid disease, hypersensitivity to retinoids or hypervitaminosis A. Patients who had vital signs or clinical laboratory findings outside specified limits, had an allergy to soya; had marked baseline prolongation of QT/QTc intervals or risk factors for torsade de pointes or Wolff-Parkinson-White syndrome; had taken supplements containing ≥10,000 international units of vitamin A within 60 days of screening; had any severe acute or chronic medical or psychiatric condition, that may have increased the risk associated with study participation or interfered with the interpretation of study results were also excluded. Previous participation in a clinical trial for the treatment of RPE65-related Leber congenital amaurosis (LCA2) was also an exclusion criterion. Common among such trials (clinical trial numbers: NCT00749957, NCT01496040, NCT00516477, NCT01208389) is the use of replication deficient recombinant AAV2 vectors carrying the wildtype human *RPE65* cDNA delivered surgically to the subretinal space.
