# Supplementary material for: Safety and Proof-of-Concept Study of Oral QLT091001 in Retinitis Pigmentosa Due to Inherited Deficiencies of Retinal Pigment Epithelial 65 Protein (RPE65) or Lecithin:Retinol Acyltransferase (LRAT)
Source: PLoS One. 2015 Dec 10;10(12):e0143846. doi: 10.1371/journal.pone.0143846 (PMC4687523; doi:10.1371/journal.pone.0143846)
Supplement: S3 Text — (PDF) [file pone.0143846.s012.pdf]

S3 Text. Calculation of Functional Retinal Area.

Goldmann visual field charts were scanned and stored as .jpg files in a central database. They were sent to an independent digitizer, and 2 readers (authors AKB and GD) verified the correctness of the results. The conversion from the Goldmann visual field chart to steradians and functional retinal areas was performed according to the Methods described in Ref. 35. After opening a GVF scan on their computer screen, digitizers first entered the subject ID, eye, operator initials, test date, fixation quality (from 5 for “good fixation” to 1 for “unable to hold fixation”), and test duration. They then performed a calibration of the scan by mouse clicks on 5 cardinal points: chart center, 90° right, 70° up, 90° left, and 70° down. They then entered the points marked by the GVF operator as transitions between seeing and non-seeing retina, for each point along each contour, for each test light used, indicating before the start of each contour whether it enclosed seeing or non-seeing retina, and what test light was used. The GVF descriptors and calibration information and all mouse clicks were recorded in a log file for later verification. A separate result file contained the GVF descriptors; the area enclosed in each contour, in deg^2 of the chart, in steradians of the perimeter bowl, and in mm^2 of retina; and a set of net seeing areas for each test light used; the combined retinal area was also expressed in log mm^2.
