# Supplementary material for: Safety and Proof-of-Concept Study of Oral QLT091001 in Retinitis Pigmentosa Due to Inherited Deficiencies of Retinal Pigment Epithelial 65 Protein (RPE65) or Lecithin:Retinol Acyltransferase (LRAT)
Source: PLoS One. 2015 Dec 10;10(12):e0143846. doi: 10.1371/journal.pone.0143846 (PMC4687523; doi:10.1371/journal.pone.0143846)
Supplement: S4 Text — (PDF) [file pone.0143846.s013.pdf]

S4 Text. Details for Functional Magnetic Resonance Imaging

Subjects.

Two consecutive retinitis pigmentosa patients (110 and 111) were recruited after fMRI was introduced at the McGill Site. Patient 110 was a male subject with *LRAT* mutations, aged 28 years. Patient 111 was also a male subject with *LRAT* mutations, aged 41. Both patients were scanned on four occasions, following the same protocol each time. Two pre-treatment scan sessions and two post treatment sessions were scheduled. For patient 110, scans occurred 7 days and 5 days prior to the start of treatment, and 9 and 13 days after. For patient 111, scans occurred 4 days and 2 days prior to the start of treatment, and 10 and 11 days after. The patients provided informed consent for the experiments approved by the Research Ethics Board of the Montreal Children's Hospital of the McGill University Health Centre.

Magnetic resonance parameters for visual cortex activation.

Scanning was done in a 3T Siemens MRI scanner as the subject lay supine. The head was secured with foam pillow padding, and patients viewed stimuli comfortably while gazing up at a mirror that reflected the display screen. All sessions lasted less than one hour. During the first session we acquired 15 minutes of anatomical images that were required to generate the 3D cortical surface reconstruction used for visualizing the functional data. The subsequent sessions required only a 5 minute anatomical scan for registration of functional scans with the 3D model. Our standard 3T fMRI protocol is as follows. *Anatomical Scans*: After a localizing scan is obtained, T1-weighted magnetization-prepared rapid gradient-echo (MP-RAGE) sequence optimized for contrast between grey and white matter (176 slices, repetition time (TR) 2,300 ms, echotime (TE) 2.98 ms, FOV 256, voxel size 1 X 1 X 1 mm) is acquired. Each scan is 5'12" in length, and either 3 or 1 scan is acquired as described above. *Functional Scans*: Functional whole brain images are acquired using a T2*-weighted gradient echo, echo-planar imaging sequence (38 slices, repetition time (TR) 3000 ms, echo time (TE) 30 ms, FOV 192, voxel size 4 X 4 X 4 mm). These signals reflect neural activity via local oxygen consumption and blood flow. For all sessions, 4 functional scans were performed. Two pattern scans were performed (see below) and two other scans not reported here were conducted. During pattern scans, patients were instructed to stare at the center of the fixation cross and to pay attention to the stimuli. The four types of stimuli were shown sequentially in epochs of 16 sec duration and each repeated 4 times, for a total duration of 4 minutes and 16 seconds.

Stimuli.

Stimuli were comprised of 4 different conditions that were presented to the subject according to a blocked design, alternating in 16 second epochs. We employed four gray-scale stimulus conditions: high contrast pattern, medium contrast pattern, low contrast pattern, and a homogeneous mid-level gray background with fixation mark only. In each case the pattern consisted of a radial square-wave grating (i.e., concentric rings) expanding and contracting at a rate of 0.5 Hz. The size of the stimuli was approximately 12 by 12 degrees. The luminance

contrasts were 99%, 45%, and 9%, respectively for high, medium and low contrast. Mid level gray was approximately 30 cd/m2**.** The stimuli were similar to those used previously to study normal visual cortex [Tootell et al., 1995] and were chosen to elicit a strong response from occipital cortex, that should increase systematically with increasing luminance contrast [e.g. Tootell et al., 1998; Boynton et at., 1999].

Data analysis.

Cortical surface reconstruction. We use the FreeSurfer package. More complete details of this procedure are described elsewhere [Dale et al., 1999; Conner et al., 2007]. There are major advantages to our flat map procedure for visualization of the inherently 2D maps that remain intact when the cortical sheet is unfolded. The images used for surface reconstruction are high-resolution anatomical scans of the whole brain optimized for contrast between gray and white matter (MPRAGE). Those structural images are combined to yield a 3-dimensional reconstruction of each subject's brain, which can then be "inflated" and flattened for better data visualization.

Functional data analysis. We use the FS-fast software in conjunction with Freesurfer. Fuller details of our procedures are described elsewhere [Mendola et al., 1999; Conner et al., 2004; Conner et al., 2007]. Preprocessing consists of correction of minor head motion and intensity normalization to remove baseline drift. Analyses of stimulus-locked activity are computed and displayed in pseudocolor, scaled according to statistical significance (*f* values). The data were analyzed separately by subject to yield a contrast map for each session [$p = 0.01$ to $p = 0.0001$, corrected for multiple comparisons with the false discovery rate]. The session level maps were combined in a second-level fixed effects repeated measures analysis to test whether there was a difference between the session-level responses, before versus after treatment. This was confirmed with intersession statistics corrected for multiple comparisons using a cluster-based Monte Carlo simulation, using a cluster-forming threshold of $p < 0.01$, 10000 iterations, and a cluster cutoff of $p = 0.05$ [Hagler et al., 2006].

References:

Boynton GM, Demb JB, Glover GH, Heeger DJ. (1999) Neuronal basis of contrast discrimination. Vision Res. 39(2):257-69.

Connor IP, Odom JV, Schwartz TL, Mendola JD. (2007) Retinotopic maps and foveal suppression in the visual cortex of amblyopic adults. J Physiol. 583(Pt 1):159-173.

Dale AM, Fischl B, Sereno MI. Cortical surface-based analysis: I. Segmentation and surface reconstruction. (1999) Neuroimage. 9(2):179-194.

Hagler DJ Jr, Saygin AP, Sereno MI. Smoothing and cluster thresholding for cortical surface-based group analysis of fMRI data. Neuroimage, 2006 33(4):1093-103.

Tootell RB, Reppas JB, Kwong KK, Malach R, Born RT, Brady TJ, Rosen BR, Belliveau JW. (1995) Functional analysis of human MT and related visual cortical areas using magnetic resonance imaging. J Neurosci. 15(4):3215-30.
