# Supplementary material for: Safety and Proof-of-Concept Study of Oral QLT091001 in Retinitis Pigmentosa Due to Inherited Deficiencies of Retinal Pigment Epithelial 65 Protein (RPE65) or Lecithin:Retinol Acyltransferase (LRAT)
Source: PLoS One. 2015 Dec 10;10(12):e0143846. doi: 10.1371/journal.pone.0143846 (PMC4687523; doi:10.1371/journal.pone.0143846)
Supplement: S5 Text — (PDF) [file pone.0143846.s014.pdf]

S5 Text. List of Participating Sites

Site 1	Montreal Children's Hospital and McGill Ophthalmology<br>McGill University Health Center<br>Montreal, QC, Canada
Site 2	Rotterdam Eye Hospital and Ophthalmic Institute<br>Rotterdam, The Netherlands
Site 3	Moorfields Eye Hospital and Institute of Ophthalmology<br>University College London<br>London, UK
Site 4	Institute for Ophthalmic Research<br>Center for Ophthalmology<br>University of Tubingen<br>Tubingen, Germany
Site 5	Scheie Eye Institute<br>Philadelphia, PA, USA
Site 6	Wilmer Eye Institute<br>Johns Hopkins University<br>Baltimore, MD, USA
Site 7	Chicago Lighthouse<br>Pangere Center for Inherited Retinal Diseases<br>Chicago, IL, USA
Training and Certification<br>VA and Goldmann VF	The EMMES Corporation<br>Rockville, MD, USA
Reading Center for GVF	Johns Hopkins University<br>Baltimore, MD, USA
Contract Research Organization	PSI CRO<br>Kidlington, Oxford, UK
